# OligoTRAFTACs: A generalizable method for transcription factor degradation†

**DOI:** 10.1039/d2cb00138a

**Published:** 2022-07-26

**Authors:** Kusal T. G. Samarasinghe, Elvira An, Miriam A. Genuth, Ling Chu, Scott A. Holley, Craig M. Crews

**Affiliations:** Department of Molecular, Cellular & Developmental Biology, Yale University New Haven CT 06511 USA craig.crews@yale.edu; Department of Pharmacology, Yale University New Haven CT 06511 USA; Department of Chemistry, Yale University New Haven CT 06511 USA

## Abstract

Dysregulated transcription factors (TFs) that rewire gene expression circuitry are frequently identified as key players in disease. Although several TFs have been drugged with small molecules, the majority of oncogenic TFs are not currently pharmaceutically tractable due to their paucity of ligandable pockets. The first generation of transcription factor targeting chimeras (TRAFTACs) was developed to target TFs for proteasomal degradation by exploiting their DNA binding ability. In the current study, we have developed the second generation TRAFTACs (“oligoTRAFTACs”) composed of a TF-binding oligonucleotide and an E3 ligase-recruiting ligand. Herein, we demonstrate the development of oligoTRAFTACs to induce the degradation of two oncogenic TFs, c-Myc and brachyury. In addition, we show that brachyury can be successfully degraded by oligoTRAFTACs in chordoma cell lines. Furthermore, zebrafish experiments demonstrate *in vivo* oligoTRAFTAC activity. Overall, our data demonstrate oligoTRAFTACs as a generalizable platform towards difficult-to-drug TFs and their degradability *via* the proteasomal pathway.

## Abstract 2

Targeted transcription factor degradation using oligonucleotide-based transcription factor targeting chimeras (TRAFTACs).

## Graphical abstract

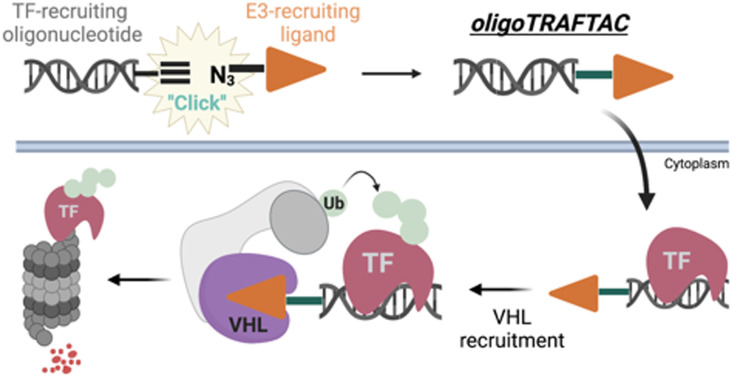

## Introduction

Protein degradation is an integral component of cellular homeostasis which supports the healthy environment within the cell. The ubiquitin-proteasomal pathway is one of the key mechanisms by which unwanted or defective proteins are degraded.^1,2^ Targeted protein degradation by PROteolysis TArgeting Chimeras, or PROTACs, has stimulated the development of novel strategies to induce protein degradation, for both discovery biology and therapeutics.^3^ PROTACs are small molecule-based heterobifunctional molecules that recruit an E3 ligase complex to a protein of interest (POI).^4^ By doing so, PROTACs induce the ubiquitination and degradation of the POI *via* the proteasome. Although PROTACs have the potential to induce the degradation of numerous proteins, the identification of small molecule recruiting ligands for several classes of proteins is still challenging and time-consuming.^5^ Therefore, the development of alternative, proximity-inducing strategies would help to target disease-causing proteins, such as transcription factors.

Transcription factors (TFs) control gene expression by binding to specific DNA elements within a given gene promoter or distal enhancer regions.^6,7^ Many diseases such as neurological disorders, autoimmunity, developmental syndromes, and many cancers result from abnormalities in the TF-controlled gene-regulatory circuitry within the diseased cell.^8–10^ For instance, the TF c-Myc, the most frequently amplified oncogene, has been extensively studied and established as a direct mediator of tumorigenesis in numerous cancers.^11^ Although indirect approaches such as bromodomain protein inhibitors and PROTACs have been explored to control c-Myc levels, direct inhibition or degradation of c-Myc has been an as yet unrealized goal.^12,13^ In contrast to c-Myc, T-box transcription factor (“brachyury”) is expressed only in a restricted set of cancer types and minimally expressed in normal cells.^14^ Brachyury is a master developmental TF that plays a pivotal role during early embryonic development in vertebrates.^15^ Brachyury expression is limited only to embryonic developmental stages and, in general, expression levels are highly downregulated in adult tissues.^16^ In addition to its regulatory functions during development, brachyury expression in remnant notochord cells in adults has been shown to be a key oncogenic driver in the rare bone cancer, chordoma.^17^ Chromosomal aberrations such as chromosome 6 gains and partial polysomy have been identified as potential molecular mechanisms in brachyury-dependent chordoma tumors.^18–21^ Other TFs such as NF-kB, STAT3/5, the androgen receptor (AR) and the estrogen receptor (ER) are other known oncogenic drivers that also rewire transcriptional circuitry in various cancer types.^22,23^

**Fig. 1:**
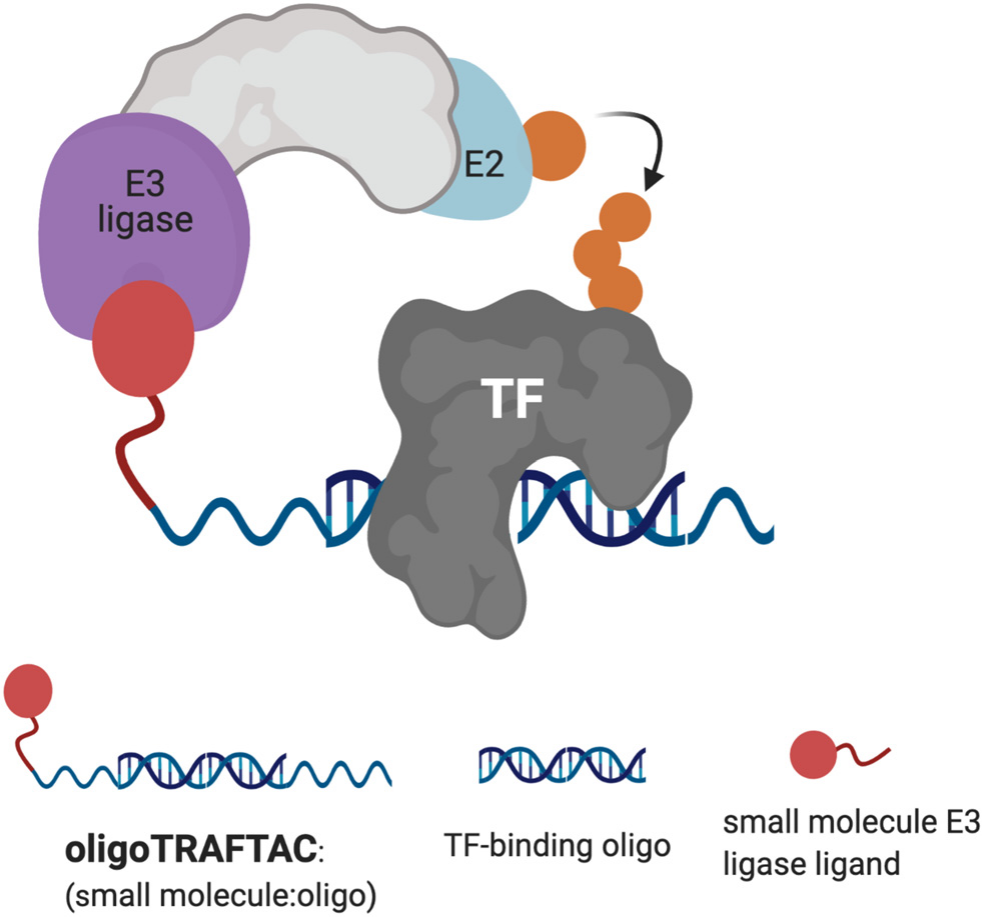
Schematic representation of oligoTRAFTAC-mediated transcription factor (TF) and E3 ligase recruitment, and proximity-dependent TF ubiquitination.

Although several TFs, such as STAT3, AR and ER have been successfully degraded by PROTACs, a plethora of disease-relevant yet traditionally “undruggable” TFs remain unaddressed.^24–26^ Recently, we developed a generalizable TF degradation platform by taking advantage of the DNA binding ability of TFs.^27,28^ In this approach, we generated a chimeric DNA:CRISPR RNA molecule – a TRAnscription Factor TArgeting Chimera (TRAFTAC) – that binds a dCas9-HaloTag7 fusion adaptor, which in turn recruits an E3 ligase into proximity with the transcription factor of interest (TOI). Using TRAFTACs, we demonstrated for the first time that TF response elements could be used in degrader design to recruit and induce the degradation of TOI, while highlighting their generalizability as well. While that strategy could be readily used as a chemical biology tool to study the biology of understudied TFs, therapeutic application of the technology is limited.

In this current report, we describe the development of second-generation TRAFTACs – “oligoTRAFTACs” that are based solely on an oligonucleotide sequence and E3 ligase-recruiting small molecule ligand to recruit TOI and the cellular ubiquitination machinery, respectively (Fig. 1). A short oligonucleotide specific to a TOI was synthesized with a terminal alkyne either at the 3′ or 5′ end. Copper-catalyzed alkyne–azide cycloaddition (CuAAC) click reaction between the alkyne-oligonucleotide and an azide-containing VHL ligand yielded chimeric oligoTRAFTACs that enabled c-Myc and brachyury degradation in HeLa and chordoma cell lines, respectively. Furthermore, based on zebrafish experiments, we demonstrate the feasibility of oligoTRAFTACs application to *in vivo* settings without the requirement for genetic modification (*i.e.*, ectopic expression) in the system.

## Results and discussion

### Degradation of c-Myc by oligoTRAFTACs

Myc transcription factors are dysregulated in a range of cancers.^29^ Even though c-Myc has been targeted by indirect approaches, development of direct-targeting methods is hindered by the paucity of ligandable pockets and their highly disordered structure.^30^ Since c-Myc binds to specific DNA sequences with a conserved E-box sequence (CACGTG), we sought to incorporate a c-Myc binding consensus DNA-sequence into the oligoTRAFTAC (OT) design.^31^ We adapted a Myc binding consensus sequence (5′ CACGTGGTTGCCACGTG 3′) taken from one of its target gene promoters.^32^ Additionally, we included a flanking sequence at both 3′ and 5′ end of the c-Myc-targeting oligonucleotide sequence to facilitate successful double strand formation of the recognition sequence while providing a flexibility for oligoTRAFTACs. First, we synthesized a c-Myc-targeting oligonucleotide sequence with an orthogonal alkyne handle on either side as a reactive moiety to append an azide-containing VHL ligand (Fig. S1A, ESI†). Copper-catalyzed alkyne–azide cycloaddition (CuAAC) click reaction was performed to synthesize two c-Myc targeting oligoTRAFTACs (OT7: VHL ligand at the 5′ end and OT10: VHL ligand at the 3′ end of the oligonucleotide) (Fig. S1B and C, ESI†). The crude mixture was analyzed by electrophoretic mobility shift assay (EMSA) to monitor the reaction progress (Fig. S2A, ESI†). The single stranded oligoTRAFTACs were then purified by HPLC, dried and reconstituted in water. To generate double stranded oligoTRAFTACs, we then performed an annealing reaction in the presence of the reverse complementary oligonucleotide by heating to 95 °C and slowly cooling to room temperature over a 1.5 h period. In addition, we also synthesized a biotinylated version of the same c-Myc-targeting oligonucleotide and generated the double stranded version using the same annealing reaction conditions.

**Fig. 2:**
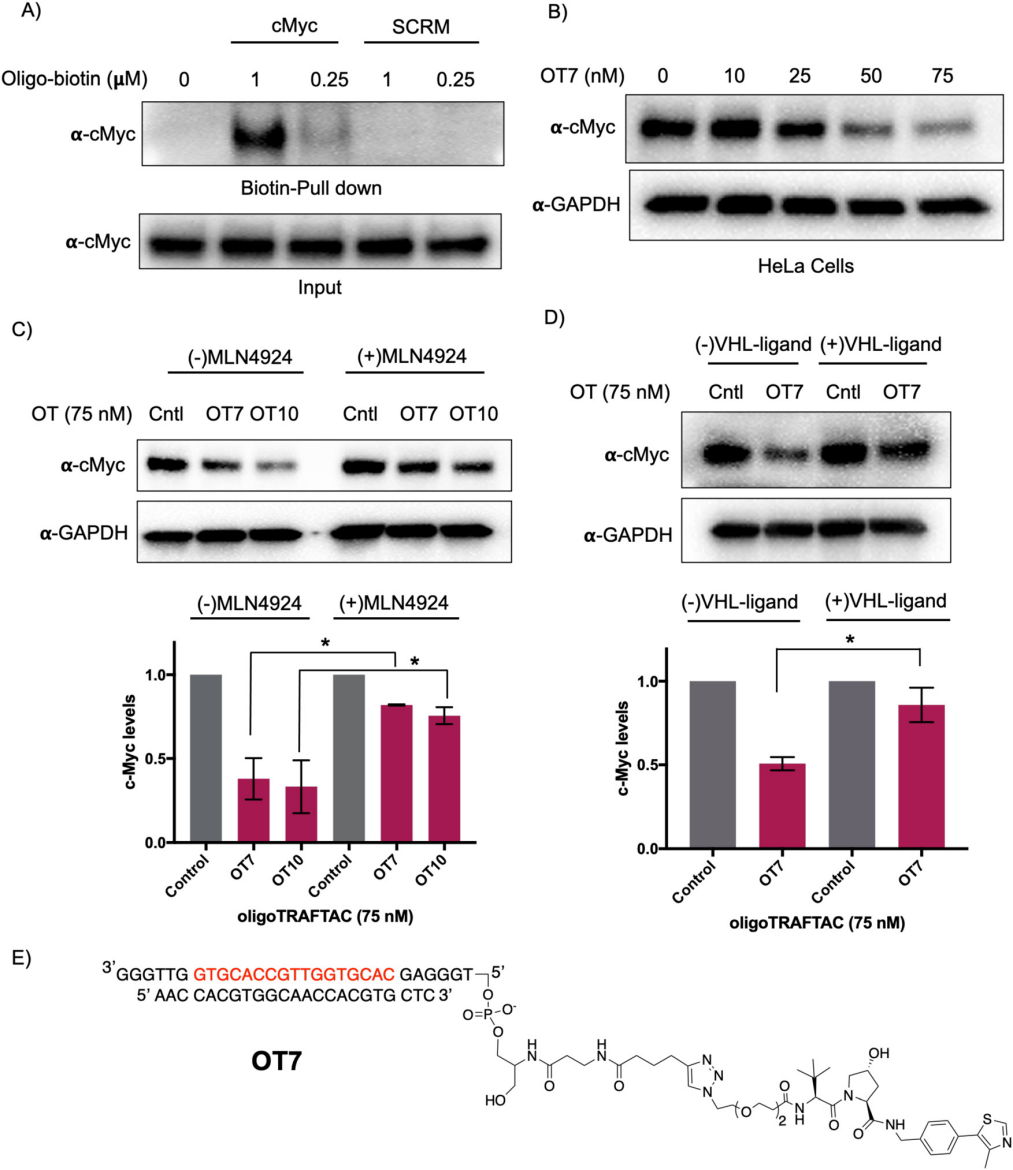
OligoTRAFTAC induces c-Myc degradation. (A) The oligonucleotide selected for the c-Myc oligoTRAFTAC engages c-Myc. HeLa cell lysates were incubated with biotinylated oligonucleotide, or its scrambled sequence, followed by capture with streptavidin agarose and probing for c-Myc. (*n* = 2) (B) Dose response of OT7-mediated c-Myc knockdown in HeLa cells. (*n* = 2) (C) OligoTRAFTAC-induced c-Myc degradation occurred *via* the proteasomal pathway. HEK293T cells were treated with c-Myc-targeting oligoTRAFTAC with and without the neddylation inhibitor, MLN-4924 (1 μM), and then analyzed for c-Myc levels. (*n* = 2, *p < 0.05) (D) HEK293 cells were preincubated with and without 10 μM VHL ligand followed by OT7 transfection and analyzed for c-Myc levels. (*n* = 2, *p < 0.05) (E) Chemical structure of the c-Myc-targeting oligoTRAFTAC (OT7).

First, we performed a biotin-pulldown experiment with the c-Myc-targeting biotin-oligonucleotide and scrambled control to confirm that the selected oligonucleotide sequence binds c-Myc. After incubation of biotin probes with HeLa cell lysate and their capture using streptavidin agarose, the eluates were probed with c-Myc antibody (Fig. 2A). The data indicated successful c-Myc engagement with the biotin-oligonucleotide probe compared to its scrambled version, thereby predicting c-Myc recruitment by the oligoTRAFTAC. We next set out to test c-Myc degradation upon transfection of increasing concentration of OT7 into HeLa cells. After 20 h post transfection, western blot analysis of the cell lysates indicated that OT7 can induce significant c-Myc degradation at 50 nM (Fig. 2B). A similar pattern was observed in HEK293T cells in response to OT7 transfection (Fig. S2B, ESI†). To test the kinetics of oligoTRAFTAC-mediated c-Myc degradation we performed a post-transfection washout experiment. After 6 h or 12 h post-transfection, cell culture medium was replaced with fresh medium and incubated for a total of 20 h. When cells were transfected for 6 h prior to washout, we observed relatively lower c-Myc degradation compared to the cells transfected for 12 h (Fig. S2C, ESI†). Furthermore, the degradation was maintained for 24 h (total of 36 h) post-washout indicating a possible catalytic behavior of oligoTRAFTACs (Fig. S2D, ESI†).

After confirming that c-Myc is susceptible to oligoTRAFTAC-mediated degradation, next we tested whether the observed degradation occurs through the proteasomal pathway. To address this, we evaluated whether neddylation inhibition, which disrupts cullin RING E3 ligase function, affects the activity of the c-Myc-targeting oligoTRAFTACs. When cells were pre-incubated with MLN-4924, c-Myc degradation was significantly diminished, demonstrating that OT7 and OT10-mediated c-Myc degradation is neddylation-dependent and occurs *via* the proteasomal pathway (Fig. 2C). Next, we performed a VHL ligand competition experiment to confirm that oligoTRAFTAC-mediated c-Myc degradation is VHL-dependent. Cells were preincubated in the presence or absence of excess VHL ligand for 1.5 h before transfecting oligoTRAFTACs for 20 h. As anticipated, we observed c-Myc levels in the VHL ligand-incubated cells were maintained relative to the cells that were not incubated with VHL ligand (Fig. 2D). Overall, the data indicated that OT7 and OT10 induces c-Myc degradation *via* the proteasomal pathway. To test the effect of c-Myc degradation on cell proliferation, we assessed the cell viability in response to the transfection of OT7 and OT12 (scrambled oligoTRAFTAC). As the data indicate, the induction of OT7-mediated c-Myc degradation inhibited cell proliferation compared to scrambled control-transfected cells (Fig. S2E, ESI†).

### OligoTRAFTAC-mediated brachyury degradation

T-box transcription factor (brachyury) is a DNA-interacting TF that regulates gene expression during early embryonic development in vertebrates. Brachyury is crucial for early mesoderm formation and plays a key role in notochord development where, as a homodimer, it recognizes and binds to a consensus palindromic DNA sequence.^33–35^ To investigate brachyury degradation by oligoTRAFTACs, we adapted a brachyury-binding DNA sequence (AATTTCACACCTAGGTGTGAAATT) as the brachyury-recruiting element in our new oligoTRAFTACs design.^36^ We synthesized oligonucleotides with flanking bases and terminal alkyne moieties at either end (3′ and 5′) of the oligonucleotide (Fig. S1A, ESI,† right panel).

**Fig. 3:**
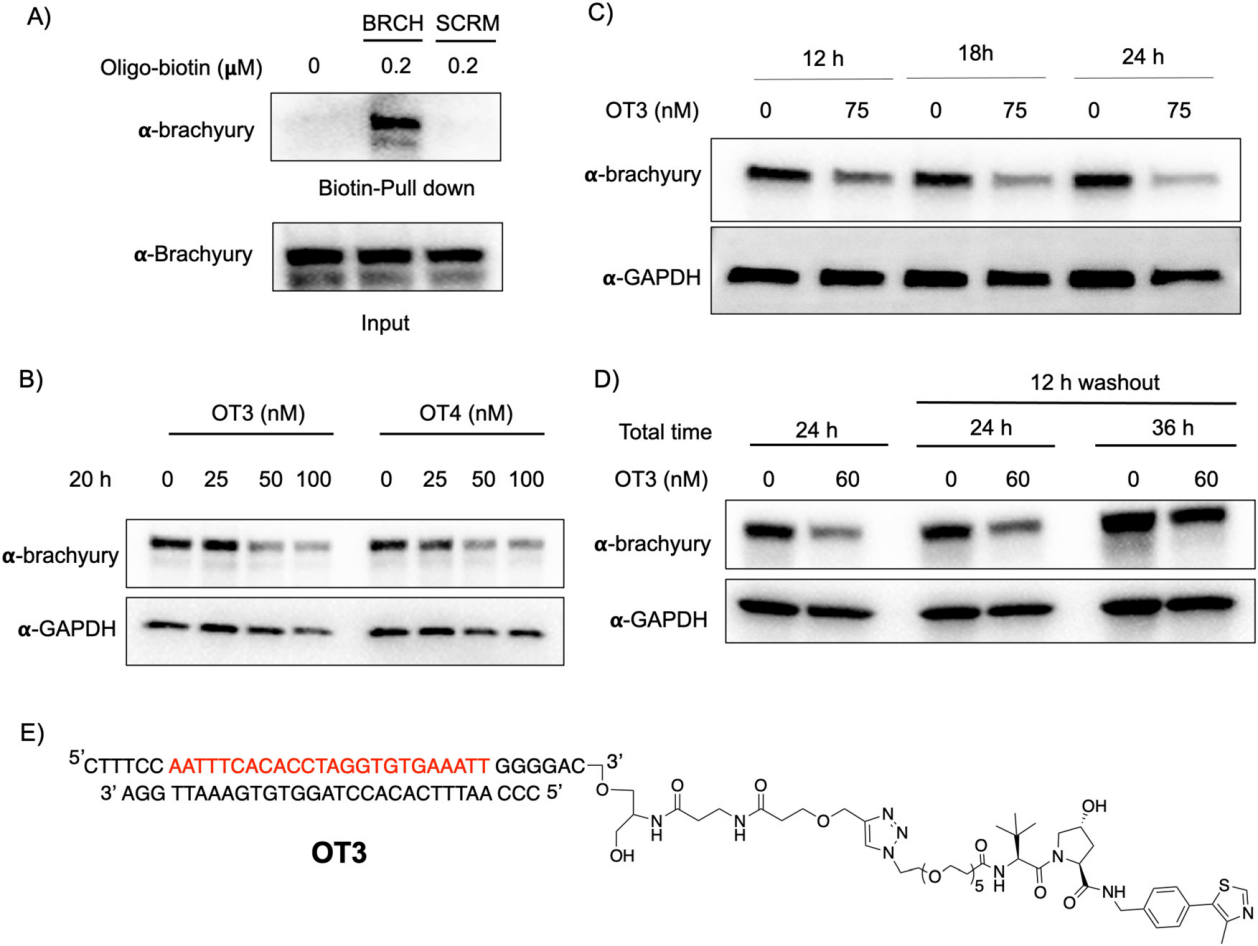
Brachyury-GFP degradation by oligoTRAFTACs. (A) Brachyury-targeting oligonucleotide used in the oligoTRAFTAC design engaged with brachyury-GFP. Brachyury targeting biotinylated oligonucleotide (BRCH) or its scrambled oligonucleotide (SCRM) incubated with cell lysate and captured by streptavidin agarose beads. (*n* = 2) (B) Two oligoTRAFTACs with 3′ VHL ligand modifications, OT3 (5 PEG unit linker) and OT4 (2 PEG unit linker) were transfected into HEK293T cells and brachyury-GFP levels were analyzed in lysates prepared after 20 h. (*n* = 2) (C) OT3 induced brachyury-GFP degradation as early as 12 h in HEK293T cells. (*n* = 2) (D) Washout experiment after 12 h of OT3 transfection. Cells were incubated continuously for 24 h in transfection medium or OT3 was aspirated after 12 h of transfection and fresh medium added to cells. Washout cells incubated for another 12 h and 24 h prior to harvesting. (*n* = 2) (E) Chemical structure of brachyury-targeting oligoTRAFTAC (OT3). OT3 consists of a phosphodiester backbone.

To generate brachyury-targeting oligoTRAFTACs, we synthesized two azido-VHL ligands with a long (5 PEG units) and a short (2 PEG units) linker (Fig. S1C and S3A, ESI†). We tested whether the oligonucleotide used in oligoTRAFTAC design could recruit brachyury by performing a streptavidin pull-down experiment using a biotin-oligonucleotide. After incubating the brachyury targeting biotin-oligonucleotide or its scrambled version for 1.5 h with the lysate of HEK293T cells that stably express a brachyury-GFP fusion, streptavidin beads were added to cell lysates and incubated for another 16 h at 4 °C to capture the binary complex. After several washings, eluted fractions were analyzed by western blotting. Biotin pulldown data indicated that the oligonucleotide used in brachyury-targeting oligoTRAFTAC design is engaged with its target protein (Fig. 3A). In contrast, scrambled oligonucleotide failed to bind brachyury-GFP, indicating sequence-specific brachyury recruitment.

To test whether a brachyury-targeting oligoTRAFTAC induces degradation, brachyury-oligoTRAFTACs were transfected into brachyury-GFP-expressing HEK293T cells for 24 h, after which cells were collected and lysed, followed by western blotting. OligoTRAFTACs with 5′ VHL-ligand and a longer linker (OT1) did not induce significant degradation of brachyury-GFP (Fig. S3B, ESI†), while its counterpart the shorter linker (OT2) successfully induced target degradation. However, oligoTRAFTACs with 3′ VHL-ligand (OT3 and OT4) both showed comparable, better degradation profiles relative to OT1 and OT2 (Fig. S3B, ESI† last two panels). Next, different concentrations of OT3 and OT4 were transfected into HEK293T cells and lysed after 20 h. Data indicated that both OT3 and OT4 could induce significant brachyury-GFP degradation at 50 nM concentration within 20 h of transfection (Fig. 3B). Furthermore, time course experiment suggested that OT3 can induce significant brachyury-GFP degradation at 12 h that is maintained up to 36 h post-transfection (Fig. 3C and Fig. S4A, ESI†). Next, we performed a washout experiment to determine the minimum incubation time to induce a noticeable brachyury degradation by OT3. To address this question, we transfected 75 nM of OT3 into HEK293T cells expressing brachyury-GFP followed by replacement of the transfection medium after 6 h and 12 h with fresh cell culture medium (Fig. S4B, ESI†).

Degradation data indicated that OT3 incubated for at least 12 h induces significant brachyury degradation comparable to continuous 24 h incubation, whereas OT3 incubation for 6 h did not induce significant brachyury degradation (Fig. 3D, center panel). Furthermore, brachyury degradation was less prominent after cells were incubated in fresh media for a longer time (36 h) (Fig. 3D, the last panel) indicating a progressive loss of brachyury degradability by OT3. This could be partially explained by the reduced stability of oligoTRAFTACs due to the oligonucleotide sensitivity towards intracellular nucleases, increased deubiquitinase (DUB) activity, or increased brachyury resynthesis.

**Fig. 4:**
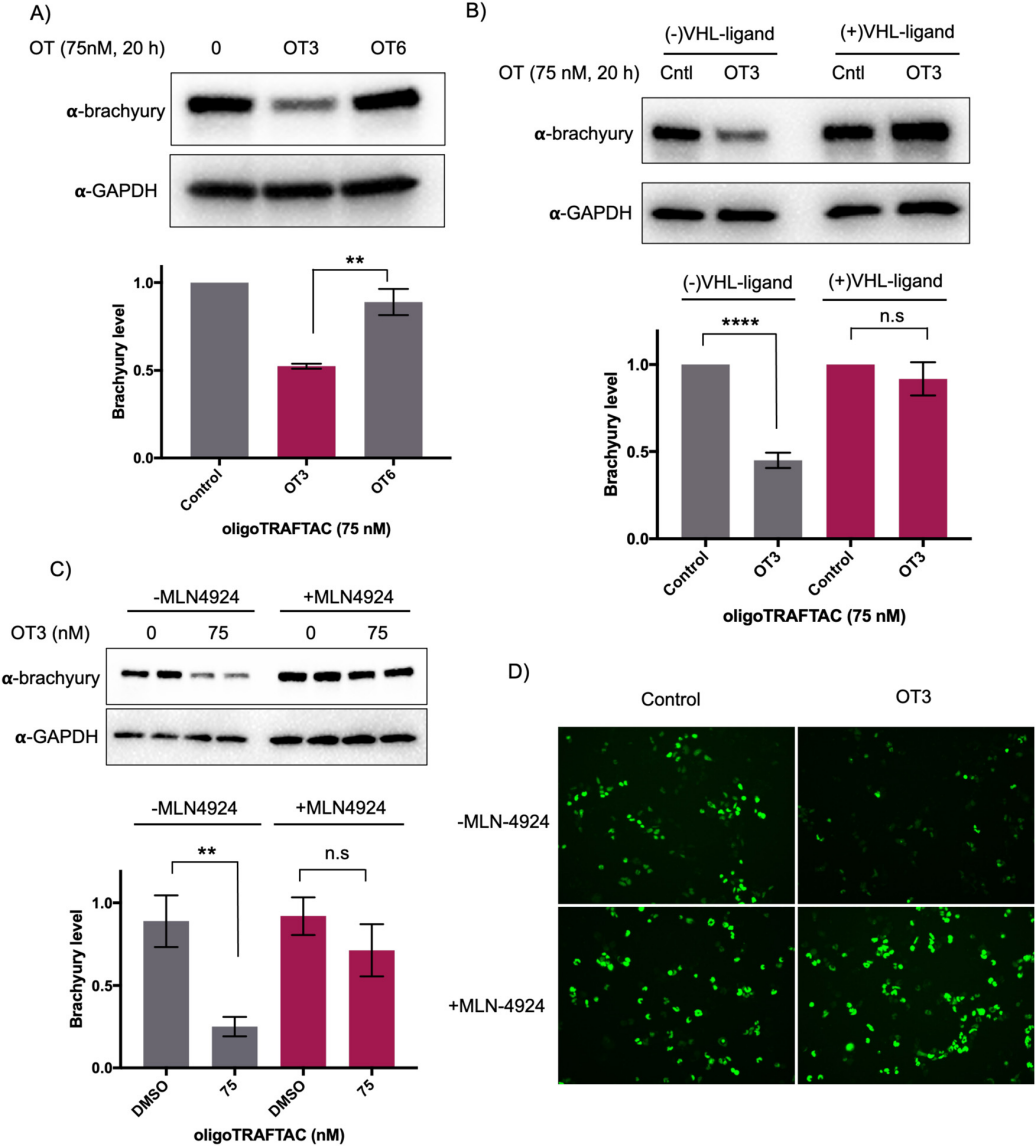
OligoTRAFTACs induce brachyury-GFP degradation *via* the proteasomal pathway. (A) HEK293T cells expressing brachyury-GFP were transfected with 75 nM each of OT3 and its scrambled OT6, and lysates were probed for brachyury levels. (*n* = 2, **p < 0.005) (B) OT3 induced brachyury degradation is VHL-dependent. HEK293T cells were preincubated with and without 10 μM of VHL ligand for 1.5 h prior to OT3 transfection. After 20 h of transfection, cells lysates were prepared and analyzed for brachyury degradation. (*n* = 3, ****p < 0.0001) (C) OT3 induces brachyury degradation *via* the proteasomal pathway: neddylation inhibitor MLN-4924 was preincubated with cells prior to OT3 transfection. After 20 h of transfection of OT3, cells were harvested and analyzed for brachyury levels. (*n* = 2, **p < 0.01) (D) Brachyury-GFP downregulation was monitored by GFP fluorescence in cells in the presence or absence of MLN-4924. (*n* = 2).

To test whether the oligoTRAFTAC-mediated brachyury recruitment and degradation is oligonucleotide-dependent, we synthesized and evaluated a scrambled-oligoTRAFTACs (OT5 and OT6) for brachyury degradation in cells. Consistently, OT3 induced a robust brachyury-GFP degradation, whereas neither OT5 nor OT6 induced significant brachyury degradation (Fig. 4A and Fig. S5, ESI†). We next performed a VHL ligand competition experiment to confirm brachyury degradation is dependent on VHL. In this experiment, cells were pretreated with 100-fold excess VHL ligand prior to OT3 treatment. As anticipated, VHL competition rescued oligoTRAFTAC-mediated brachyury degradation (Fig. 4B), confirming that observed brachyury degradation is a result of VHL E3 ligase recruitment by OT3. Furthermore, we evaluated OT3-mediated brachyury degradation in the presence of a neddylation inhibitor to further confirm that the intended mechanism is *via* the proteasomal pathway. Similar to the VHL competition experiment, the neddylation inhibitor, MLN-4924 was pre-incubated with cells at 1 μM concentration. After 1.5 h, OT3 was transfected into the cell for 20 h following which the cell lysates were analyzed as indicated in Fig. 4C. The data indicated that OT3 could not induce brachyury degradation in the presence of MLN-4924, showing that neddylation is crucial for the mechanism of action (Fig. 4C). Furthermore, analysis of GFP-fluorescence confirmed brachyury degradation only in the absence of MLN-4924 (Fig. 4D). Overall, these data support the fact that OT3-induced brachyury degradation is mediated through the proteasomal pathway and is dependent on the oligonucleotide sequence of OT3.

### OligoTRAFTACs induce brachyury degradation in chordoma cells

**Fig. 5:**
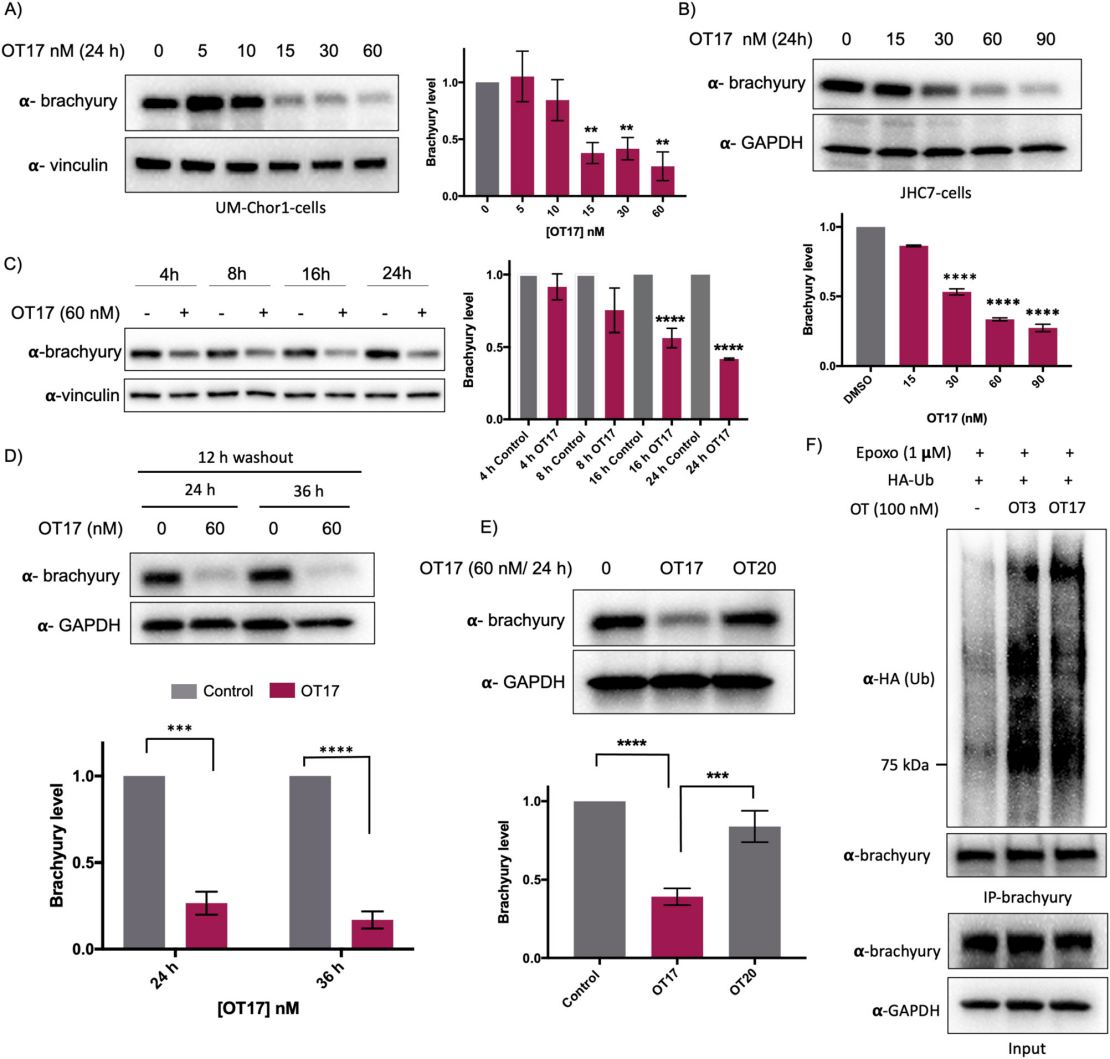
Endogenous brachyury degradation by oligoTRAFTACs constructed with phosphorothioate backbone. (A) Increasing concentrations of OT17 were transfected into UM-Chor1 cells and harvested after 24 h, subjected to lysis and analyzed for brachyury downregulation. Brachyury levels were normalized to loading control and presented as a bar graph. (*n* = 2, **p < 0.01) (B) JHC-7 cells were transfected with OT17 and probe for brachyury levels (*n* = 2, ****p < 0.0001). (C) UM-Chor1 cells were transfected with 60 nM of OT17 and harvested at subsequent different time points as indicated. (*n* = 3, ****p < 0.0001) (D) Washout experiment: transfection medium was removed after 12 h of OT17 transfection and UM-Chor1 cells were incubated for another 12 h or 24 h in fresh complete cell culture medium. (*n* = 2, ***p < 0.001, ****p < 0.0001) (E) OT17-mediated brachyury degradation is oligonucleotide sequence dependent. UM-Chor1 cells were transfected with OT17 and scrambled OT20, cells were lysed and analyzed as shown. (*n* = 2, ***p < 0.001, ****p < 0.0001) (F) OT3- and OT17-induced brachyury ubiquitination. HEK293T cells that overexpress brachyury-GFP were transfected with HA-ubiquitin, followed by the second transfection with OT3 or OT17. After 12 h, cell lysates were subjected to immunoprecipitation using brachyury antibody, and the eluates blotted for the indicated proteins. (*n* = 3).

Increasing OT3 concentrations were transfected into UM-Chor1 cells followed by western blotting to determine brachyury degradation. Consistent with the brachyury-GFP degradation in HEK293T cells, 60 nM of OT3 induces ∼70% brachyury degradation in UM-Chor1 cells 24 h post-transfection (Fig. S5B, ESI†). Although OT3 induced comparable levels of brachyury degradation in both cell lines, phosphodiester linkages within OT3 are susceptible to cleavage by both extra- and intracellular nucleases. Therefore, we next synthesized oligoTRAFTACs with a phosphorothioate (PS) backbone.^37^ The addition of extra non-bridging sulfur atoms in the inter-nucleotide phosphate group has been shown to have both increased stability against nucleases and improved cell permeability.^37^ A brachyury-targeting oligoTRAFTAC, OT17, was synthesized by incorporating PS bonds throughout the oligo sequence. Increasing OT17 concentrations were transfected into chordoma cells (UM-Chor1) for 24 h following which the cells were lysed and probed for brachyury levels – western blotting showed that OT17 induced significant degradation of endogenous brachyury even at 15 nM (Fig. 5A). We noticed a similar degradation pattern in another chordoma cell line, JHC-7 (Fig. 5B), although OT17 was slightly less potent in them, requiring 30 nM to induce brachyury knockdown comparable to UM-Chor1 cells.

Time course experiment showed that OT17 induced a modest degradation at 8 h, which increased to ∼60% knockdown 16 h post-transfection (Fig. 5C) although washout experiment indicated that incubation of transfection complex for 12 h is sufficient to induce significant brachyury degradation (Fig. 5D). Furthermore, the same experiment displayed persistent degradation for 24 hours post washout (a total of 36 h post-transfection), suggesting increased stability of OT17 compared to OT3. Furthermore, the data suggest a possible catalytic mechanism for OT17. We also synthesized and tested a scrambled oligoTRAFTAC with a PS backbone throughout the oligonucleotide sequence (OT20). Consistently, a side-by-side comparison of brachyury levels in UM-Chor1 cells transfected with OT17 and OT20 indicated sequence-specific brachyury degradation (Fig. 5E). To monitor oligoTRAFTAC-mediated brachyury ubiquitination resulting from induced proximity to VHL, we next transfected HA-ubiquitin into HEK293 cells overexpressing brachyury-GFP. After cells were pre-incubated with proteasome inhibitor epoxomicin (500 nM), OT3 and OT17 were transfected and incubated for 12 hours. Subsequent immunoprecipitation data indicated that OT3 and OT17 could induce ubiquitination of brachyury-GFP, confirming that degradation by oligoTRAFTAC follows a ubiquitination event mediated by recruited VHL (Fig. 5F). Furthermore, the proteasome inhibition experiment indicated that OT17-mediated brachyury degradation occurred *via* the proteasome (Fig. S5C, ESI†).

### *In vivo* activity of oligoTRAFTACs: OT17-mediated developmental defects in zebrafish tail formation

**Fig. 6:**
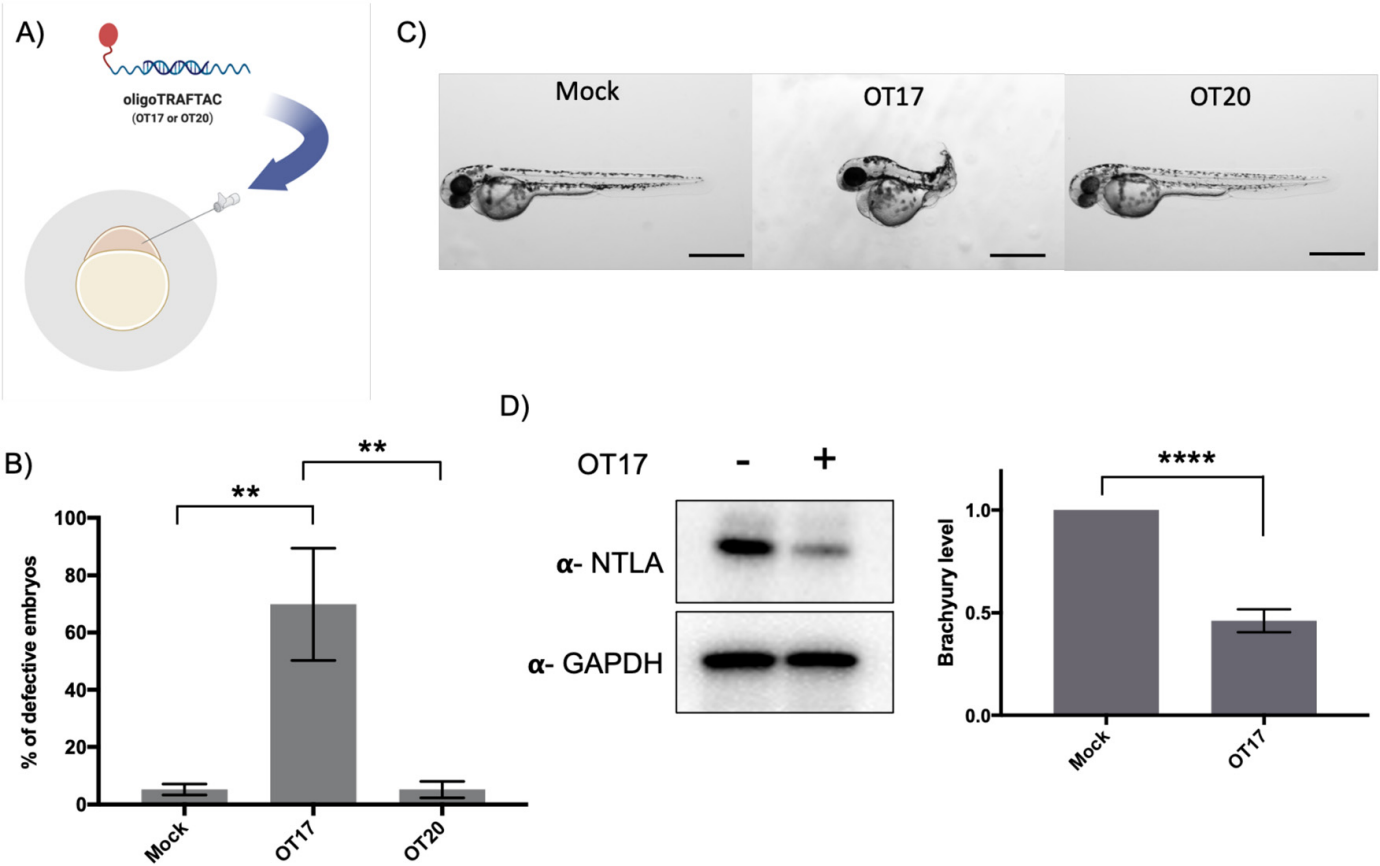
Microinjection of brachyury-targeting oligoTRAFTAC into zebrafish embryos: demonstration of *in vivo* activity. (A) Schematic representation of OT17 and OT20 microinjection into zebrafish embryos. (B) Quantitation of the defective embryos in mock, OT17 and OT20 injected groups. Mock, OT17 and OT20 (180 picoliters from 25 μM of oligoTRAFTACs, or mock equivalent) were microinjected into embryos (number of embryos in each group for three independent experiments; mock-47, 50, 43; OT17-49, 52, 61; OT20-75, 74, 45). After 48 h, the number of defective tails in each group was recorded and presented as percentage in a bar graph. (*n* = 3, **p < 0.001) (C) Images of representative zebrafish from the cognate treatment groups. Pictures were captured after 48 h post microinjection of mock, OT17 and OT20. Scale bar 500 μm. (*n* = 3) (D) Brachyury levels in zebrafish embryos after OT17 (180 picoliters from 25 μM of oligoTRAFTACs, or mock equivalent) injection. Embryos were collected at 8–10 somite stage, subjected to lysis, and probed for brachyury levels. (*n* = 3, ****P < 0.0001).

After confirming that brachyury-targeting oligoTRAFTACs induce efficient endogenous brachyury degradation in cells, we next evaluated their activity in animals using zebrafish as a model organism. Although brachyury overexpression in adult tissue is one of the key factors that leads to tumorigenesis in chordoma, brachyury is widely known for its essential biological activity in vertebrate notochord formation at early stages of embryonic development.^38^ To test for oligoTRAFTAC *in vivo* activity, we examined tail deformation in OT3-injected zebrafish embryos relative to mock-injected embryos (Fig. 6A). Interestingly, although OT3 could induce brachyury degradation in cells, OT3 did not induce tail deformation in zebrafish, possibly due to the sensitivity of its phosphodiester backbone to nucleases. However, microinjection of OT17, in which PS linkages provide stability against both exonucleases and endonucleases, induced tail deformation in ∼70% of injected embryos (Fig. 6B and C) whereas <5% mock and scrambled OT20 injected embryos had defective tails. This result illustrates how the observed brachyury loss of function phenotype is sequence-dependent, and demonstrates *in vivo* oligoTRAFTAC activity in zebrafish. In addition, we have also tested brachyury levels at 8–10 somite stage following OT17 injection. Consistent with the observed phenotype, OT17 could induce brachyury degradation during the early stage of embryonic development and subsequently affect the tail formation (Fig. 6D).

## Discussion

The majority of transcription factors, key mediators of gene expression, are considered undruggable. In this study, we developed a method for oligonucleotide-dependent TF recruitment and degradation by the proteasomal pathway. We have coupled TF-binding short DNA sequences from target gene promoters with VHL ligands to create bifunctional molecules for the targeted degradation of those same TFs. Since the binding sequences already have been identified for many TFs, and since synthetic routes for oligonucleotide synthesis are well established and economical, these oligonucleotide sequences can be rapidly employed in oligoTRAFTAC design to use as a versatile tool for both basic discovery biology and therapy development.^39^ We previously developed a strategy (first generation TRAFTACs) based on an oligonucleotide and E3 ligase recruiting intermediate protein, dCas9-Halotag7, by exploiting its ability to recruit both TOI and E3 ligase in the presence of TRAFTAC and a concurrently administered HaloPROTAC.^27^ Following the first generation TRAFTAC study, a couple of other studies were published describing the use of oligonucleotides for TF degradation.^40,41^ In addition to TF degradation, a separate study was reported showing the applicability of the oligonucleotide-based PROTAC platform for RNA-binding proteins as well.^42^

Although first generation TRAFTACs are widely applicable as chemical biology tools for discovery biology, therapeutic applicability needed further improvements. In this current study, we discuss the development of second generation TRAFTACs by replacing the intermediatory E3 ligase recruiting dCas9-Halotag7 in the first generation of TRAFTACs with a VHL-recruiting small molecule ligand. OligoTRAFTACs targeting c-Myc TF displayed a robust degradation. Addition of VHL ligand to either side of the oligonucleotide did not significantly alter its ability to induce c-Myc degradation. This can be partially attributed to the flexibility of oligoTRAFTACs provided by the extra flanking nucleotides between the VHL ligand and TF-recruiting oligonucleotide. Other oligoTRAFTACs used in this study induced the degradation of ectopically expressed brachyury-GFP and endogenous brachyury *via* the proteasomal pathway. Although washout experiments indicated that oligoTRAFTACs are not as kinetically efficient as conventional PROTACs, the transient binding nature of oligoTRAFTACs to both TF and VHL proteins suggests the probability of a similar catalytic degradation mechanism. Moreover, to our knowledge the current study provides the first evidence of the degradability of untagged, endogenous brachyury in multiple chordoma cell lines. In the first attempt to test oligoTRAFTAC *in vivo* activity, OT3 (containing a phosphodiester backbone) could not induce the intended defective tail phenotype in zebrafish. It is noteworthy that first generation TRAFTACs, where oligonucleotides are partly protected by the ribonuclearcomplex, induced defective tails in zebrafish. Therefore, a potential reason for the failure of OT3 to induce the intended phenotype within the embryos may be their high sensitivity and exposure of oligoTRAFTACs to embryonic nucleases present at the early stages of development. Therefore, phosphodiester oligoTRAFTACs might not endure *in vivo* at the intracellular concentrations needed to achieve a significant brachyury degradation and the intended phenotype. Previous studies suggested that synthesis of oligonucleotide-based entities with a phosphorothioate backbone largely benefitted from nuclease resistance. Therefore, the microinjection of phosphorothioate backbone-containing OT17 resulted in a significantly high occurrence rate of the defective tail phenotype compared to the mock or scrambled oligoTRAFTAC (OT20), confirming the increased stability and resistance of OT17 to nucleases.

It has been shown that multiple TFs recognize and bind to similar DNA sequences.^43^ Since TFs, such as TCF3, also recognizes a similar E-box consensus sequence as c-Myc, the evaluation of TCF3 degradation indicated a sequence-dependent off-target activity of OT7 (Fig. S2F, ESI†).^43^ To address the sequence specificity of oligoTRAFTACs, we tested NF-kB (p65) protein levels in response to brachyury-targeting OT17. We observed a consistent brachyury degradation in OT17-treated cells. However, since p65 does not recognize brachyury-binding DNA sequence, OT17 did not induce p65 degradation. Therefore, the data suggest a sequence-dependent brachyury degradation by OT17 (Fig. S2G, ESI†). Furthermore, in the current study, we report for the first time the use of PS modified oligonucleotides in oligoTRAFTAC design to improve its *in vivo* stability and demonstrated oligoTRAFTAC activity in zebrafish. However, similar to other oligonucleotide-based drugs, the therapeutic use of oligoTRAFTACs will be significantly challenged by their size and negatively charged nucleotides.^44^ Therefore, optimization of nanocarriers should be implemented to increase the cellular uptake and endosomal escape of oligoTRAFTACs. However, due to the faster degradation profile and catalytic nature, oligoTRAFTACs may exhibit improved therapeutic benefits than other oligonucleotide-based strategies such as RNAi. Overall, oligoTRAFTACs are programmable heterobifunctional molecules comprised of a TF-binding oligonucleotide sequence and a VHL binding ligand, which induce TF degradation in cells and displayed robust *in vivo* activity. Due to the simple and modular nature of their structure, oligoTRAFTACs can be rapidly designed for many non-ligandable DNA-binding TFs both for use as a chemical biology tool as well as a potential therapeutic strategy.

## Conflicts of interest

C. M. C is founder, shareholder, and consultant to Arvinas, Inc. and Halda, LLC, which support research in his laboratory.

## Supplementary Material

CB-003-D2CB00138A-s001
