# Supplementary material for: OligoTRAFTACs: A generalizable method for transcription factor degradation
Source: RSC Chem Biol. 2022 Jul 26;3(9):1144–53. doi: 10.1039/d2cb00138a (PMC9428672; doi:10.1039/d2cb00138a)
Supplement: CB-003-D2CB00138A-s001 [file CB-003-D2CB00138A-s001.pdf]

Electronic Supplementary Material (ESI) for RSC Chemical Biology.
This journal is © The Royal Society of Chemistry 2022

Supporting Information

OligoTRAFTACs: A Generalizable Method for Transcription Factor Degradation

Kusal T. G. Samarasinghe[a], Elvira An[c], Miriam A. Genuth[a], Ling Chu[a], Scott A. Holley[a] and Craig M. Crews*[a,b,c]

[a] Department of Molecular, Cellular & Developmental Biology, Yale University, New Haven, CT 06511, USA.

[b] Department of Chemistry, Yale University, New Haven, CT 06511, USA.

[c] Department of Pharmacology, Yale University, New Haven, CT 06511, USA.

* Correspondence: craig.crews@yale.edu

Table of Contents

1. Materials and Methods ... 3
1.1 Materials and reagents ... 3
1.2 General biology methods ... 3
1.2.1 Cell culture ... 3
1.2.2 OligoTRAFTAC transfection ... 4
1.2.3 Click rection ... 5
1.2.4 Annealing reaction ... 5
1.2.5 Western blotting ... 6
1.2.6 EMSA ... 6
1.2.6 Biotin pull down ... 7
1.2.7 Immunoprecipitation and ubiquitination assay ... 7
1.2.8 Cell viability assay ... 8
1.2.9 Zebrafish Husbandry and Microinjection ... 8
2. Supporting Figures ... 9
3. General chemistry methods ... 15
3.1 Synthesis of LC-HO-2100 ... 15
3.2 Synthesis of LC-HO-2113 ... 16
3.3 1H NMR and 13C NMR for LC-HO-2100 ... 16
3.4 1H NMR and 13C NMR for LC-HO-2113 ... 20
4. Mass spectra for oligonucleotides ... 22
5. Oligonucleotide sequences ... 25
6. Original blots ... 28
7. References ... 37

1. Materials and Methods

1.1 Materials and reagents

All cell culture media and fetal bovine serum (DMEM, IMDM, RPMI, DMEM/F-12) were purchased from Gibco unless otherwise specified. Primary antibodies for GAPDH (2118S), Vinculin (13901S), brachyury (81694S), HA-tag (3724S), protein A magnetic

beads (73778S) and streptavidin magnetic beads (5947S) were purchased from Cell Signaling Technologies. Primary antibody for c-Myc (sc-40) was purchased from Santa Cruz. Secondary rabbit (NA934) and mouse (NA931) antibodies were purchased from GE Health Care. RNAiMAX (13778-150) transfecting reagent was purchased from Thermo Fisher Scientific. MLN4924 (S7109) was purchased from Selleckchem. Anti-NTLA (SAB2702416), Copper (II) sulfate pentahydrate (209198) and L-Ascorbic acid (A4403) were purchased from Millipore Sigma, and THPTA purchased from Lumiprobe (H4050). All the oligonucleotide modifiers were purchased from Glen Research, 3' Alkyne (20-2992-41), 5' Alkyne (10-1992-90) and biotin (10-5950-90). All the oligonucleotides were custom synthesized by Yale Keck oligo synthesis facility.

1.2 General biology methods

1.2.1 Cell culture

Human embryonic kidney cells HEK293T cells and HeLa cells were grown in Dulbecco's Modified Eagles Medium (DMEM) containing 10% heat inactivated fetal bovine serum (FBS), streptomycin (5 µg/mL) and 5 U/mL penicillin 95 U/mL). All cell lines were maintained and cell culture experiments were carried out in humidified incubators at 37 degrees and 5% CO_2 supplementation.

1.2.2 OligoTRAFTAC transfection

One day prior to oligoTRAFTACs transfection, cells (HEK293T: $0.7X10^6$/well, HeLa: 0.2 $X10^6$/well, UM-Chor1: $0.2X10^6$/well and JHC7: $0.4X10^6$/well) were propagated into 6-well plates containing appropriate complete growth medium. Prior to transfection, complete medium was replaced with 1.75 mL of transfection medium (2%FBS, no Penstrep). Chimeric oligoTRAFTAC transfection was performed using RNAi-Max reagent according to the protocols provided by the manufacture. All transfections were carried out in 6-well

plates with 2 mL of media and concentrations of oligoTRAFTACs were calculated according to this volume (2 mL). Briefly, for 50 nM concentration, 4 µl from a 25 µM oligo TRAFTAC stock was added to a tube containing 125 µl of OPTIM-MEM and 12.5 µl of RNAi-Max reagent was added to a separate tube containing 125 µl of OPTIM-MEM (added ~4 µg of oligoTRAFTAC to 2 mL cell culture medium). Two tubes were incubated for 5 minutes at room temperature and oligoTRAFTAC containing OPTI-MEM was then slowly added to the second tube with RNAi-MAX. The solution in the tube was mixed well by pipetting up and down several times. After incubating for 10 minutes at room temperature, 250 µl of oligoTRAFTAC:RNAi-MAX complex was added drop wise onto cells containing the transfection medium. Transfection medium was mixed well before transferring the 6-well plate into the incubator. After appropriate time, cells were either harvested or transfection medium containing oligoTRAFTAC:RNAi-MAX complex was replaced with fresh medium and incubated for desired time point prior to harvesting. For MLN-4924 and VHL ligand competition assay, these molecules were pre-incubated in the transfection medium (1.75 mL) for 1 h prior to transfection of oligoTRAFTACs. For proteasome inhibition assay, 10 µM of MG-132 or 0.75 µM of epoxomicin was incubated with cells after 12 h of transfection of OT17. Then, cells were incubated for another 12 h before lysing cells. Cell lysates were prepared by incubating cells in RIPA lysis buffer (25 mM Tris pH 7.6, 150 mM NaCl, 1% NP40, 1% deoxycholate, 0.1% SDS, 1X protease inhibitor cocktail from Roche and 1 mM of PMSF) on ice for 30 minutes and cell lysate was clarified by centrifugation at high speed (15 000 rpm) for 20 minutes. Clear supernatant was collected for further experiments. For cell proliferation/viability assays, cells were split (HeLa: 0.1 X10^5/well) into white, clear bottom 96-well plates. Transfection was carried out as described above, except volumes, i.e., 175 µl of transfection medium per one well, 0.5 µl of RNAi-MAX/well and 25 µl of OPTIMEM per well.

1.2.3 Click rection

Alkyne-modified oligonucleotides were dissolved in ultra-pure water at 500 µM concentrations and azide-modified VHL Ligands were dissolved in DMSO at 10 mM. Right

before the click reaction, fresh stock solutions of Cu (II) sulfate pentahydrate (50 mM in water), Tris(3-hydroxypropyltriazolylmethyl)amine (THPTA: 100 mM in DMSO) and sodium ascorbate (100 mM in water) were made. The click reaction was carried out in 50% DMSO solution. First, alkyne modified oligonucleotide (250 **μ**L) and azide-modified VHL Ligand (1:5 molar ratio) mixed in tube 1. Then, Cu (II) sulfate pentahydrate and THPTA was mixed first, followed by the addition of sodium ascorbate to be final molar ratio of 1:2:2. A 37-fold molar excess of Cu-THPTA complex was added to tube 1 and water and DMSO were added to get the final reaction mixture with 50% DMSO. Click reaction mixture was mixed thoroughly and flushed with inert gas (N_2) for 1 minute. Reaction mixture was then incubated at room temperature for ~ 16 h. Click reaction product was purified by reverse phase high-performance liquid chromatography (HPLC) using a C18 column. HPLC method used for oligo purification (Buffer A-5% acetonitrile, 4.25% Triethylamine acetate (TEAA) in water; Buffer B- 100% acetonitrile (ACN). The program was set with a flow rate of 5 mL/min for 150 minutes, and a gradient of ACN increasing from 0-80%.

1.2.4 Annealing reaction

FPLC purified single stranded oligo conjugated to VHL (oligo-VHL) ligand and its reverse complement oligo were dissolved in ultra-pure water. Single stranded oligo-VHL and single stranded reverse complement oligos were mixed 1:1 molar ratio (final concentrations of TRAFTACs were set to 25 μM) in 1X annealing buffer (10 mM Tris, pH 7.5, 50 mM NaCl and 1 mM EDTA) and incubated for 10 minutes in a water bath at 95 degrees Celsius. Then, the hot-plate was turned off and the samples were left in the water bath and let cool down to room temperature over 2 h. Double stranded oligoTRAFTACs were mixed by gently vortexing and aliquoted and stored at -20 degrees Celsius. Reverse complementary sequences: **OT3/17’**- 5’CCCAATTTCACACCTAGGTGTGAAATTGGA3’, **OT7/10**- 5’ AACCACGTGGCAACCACGTGCTC 3’.

1.2.5 Western blotting

Protein concentration in all the cell lysates were measured by BCA protein assay kit and equal amounts from each lysate were mixed with 4X loading dye and boiled for 5 minutes followed by 2 minutes centrifugation prior to loading into SDS-PAGE gel. Next proteins on the SDS-PAGE gel were transferred to a PVDF membrane by western blotting and the membrane was blocked with 5% milk in TBST (0.05%Tween 20) for 1 h. Primary antibodies (all Cell Signaling antibodies were diluted 1:1000, c-Myc 1:150) were prepared in TBST with 5% milk and membranes were incubated overnight at 4^0C. On the following day, membrane was washes for 15 minutes (Incubate for three times, 5 minutes each) and appropriate secondary antibodies (1:5000) were prepared in TBST with 5% milk and incubated with the membrane for 1h at room temperature (RT). Membrane was washed for 30 minutes with TBST (incubate for three times, 10 minutes each) prior to imaging.

1.2.6 EMSA

Click reaction mixtures and unreacted alkyne-modified oligonucleotides were separated in a 1.2 % agarose gel for 1 h at constant 120 mV and DNA bands were captured by the ChemiDoc system (BioRad) using SYBR safe mode.

1.2.6 Biotin pull down

Cells (HEK293T or HeLa) were grown in three T-175 flasks for 2 days. When cells reach >90% confluency, cells were harvested and washed once with 1X PBS. Cells were pooled together and resuspended in 1.5 mL immunoprecipitation buffer (25 mM Tris pH 7.4, 150 mM NaCl, 0.4% NP40, 5% glycerol, 1X protease inhibitor cocktail from Roche and 1 mM of PMSF). Cells were then incubated for 30 minutes on ice prior to centrifugation at high

speed (15,000 rpm) for 20 minutes. Equal amounts/volumes (~1 mg) of clarified lysate were transferred to individual tubes and incubated with biotinylated double stranded oligonucleotides for 2 h at RT. Pre-washed 30 **µ**l of streptavidin agarose beads were transferred to each tube and incubated overnight at 4^0C. Beads were then washed with 1X TBS for 15 minutes (three times, 5-minute incubation each time). Bound proteins were eluted with 2X loading buffer by boiling for 8 minutes. Boiled samples were centrifuged at high speed for 5 minutes and supernatant was loaded onto SDS-PAGE gel followed by western blotting.

1.2.7 Immunoprecipitation and ubiquitination assay

A HA-tagged ubiquitin plasmid (4 µg) was transfected into HEK293T cells overexpressing brachyury-GFP in a 10 cm dish. On the following day, transfected cells were split into three 10 cm cell culture dishes and incubated for 24 h prior to transfection of oligoTRAFTACs. Epoxomicin (1 µM) was preincubated with cells for 1 h and oligoTRAFTACs (mock, OT3 and OT17) were then transfected using RNAi-MAX in 5 mL/dish of transfection medium. After 12 h post transfection, cells were harvested and lysed using immunoprecipitation buffer (25 mM Tris pH 7.4, 150 mM NaCl, 0.4% NP40, 5% glycerol, 1X protease inhibitor cocktail from Roche and 1 mM of PMSF). Approximately 1.5 mg of lysate from each sample was incubated with brachyury antibody at 4^oC for 4 h. Protein A agarose beads (30 **µ**l) were added to antibody, lysates mixture and rock at 4^oC for ~ 18 h. Beads were washed with 1X TBS for three times with 5-minute incubation during each wash. Immunoprecipitated proteins were eluted by boiling agarose beads in 2X loading buffer (containing 10% ß-ME) for 8 minutes and centrifuged at high speed for 5 minutes prior to the loading into SDS-PAGE gel followed by western blot analysis.

1.2.8 Cell viability assay

Cells were split and subjected to oligoTRAFTAC transfection as described in ‘’oligoTRAFTAC transfection” method section. Following the transfection, cells were incubated for the appropriate number of days before recording the luminescence reading from the plate reader. CellTiter-Glo reagent was prepared according to the manufactures recommendation and mixed with cell culture medium with 1:1 ratio. CellTiter-Glo reagent (100 **μ**L) was added to each well and incubated for 10 minutes before taking the reading.

1.2.9 Zebrafish Husbandry and Microinjection

Tüpfel-longfin zebrafish were raised according to standard protocols approved by the Institutional Animal Care and Use Committee. Experiments were performed before sex determination in zebrafish [1]. Embryos were injected at the one cell stage with 180 picoliters of a 25 µM oligoTRAFTAC solution. They were raised for 48 hours at 28.6 °C and then scored for the presence of tail defects. While injected embryos showed severe, moderate, and mild tail defects, only those with severe and moderate tail defects were considered for quantitation [2]. For western blot analysis, approximately 20 embryos were collected at 8-10 somite stage and deyolked using deyolking buffer (without Calcium: 55 mM NaCl, 1.8 mM KCl, 1.25 mM $NaHCO_3$). Deyolked embryos were centrifuged at 1000 rcf for 30 seconds and the pellet was isolated and washed once with 1 mL of wash buffer (110 mM NaCl, 3.5 mM KCl, 2.7 mM $CaCl_2$, 10 mM Tris/Cl pH8.5). After centrifuging at 1000 rcf for 30 seconds, the pellet was lysed in 20 µl of 2X SDS-loading buffer with 10% BME before loading the SDS-PAGE gel.

2. Supporting Figures

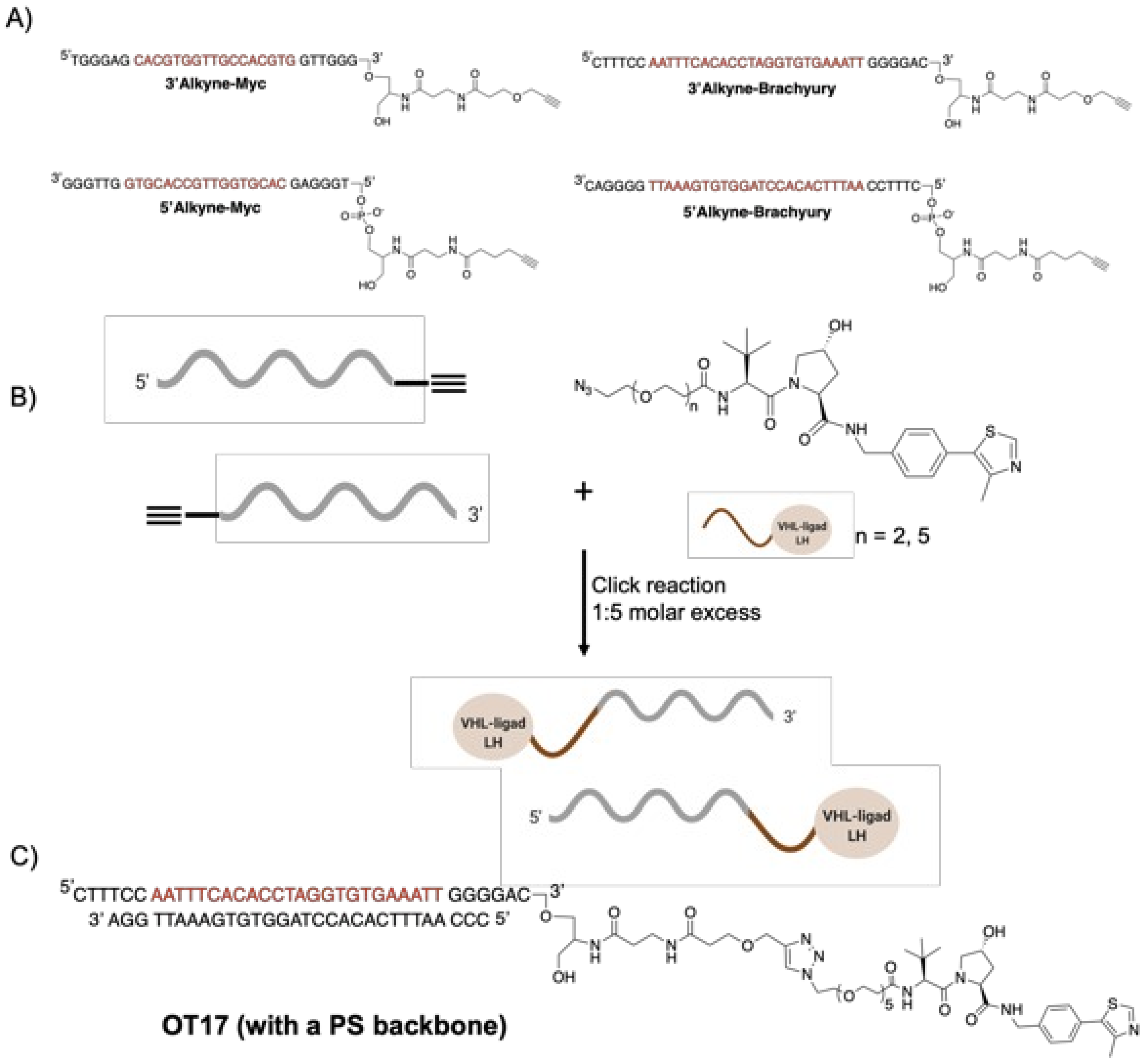

Figure S1. Oligonucleotide synthesis and click reaction. A) Oligonucleotides were custom synthesized with a terminal alkyne either at 3' or 5' end of the oligo. Oligonucleotide sequences for both 3' and 5' alkyne targeting c-Myc (left panel) and brachyury (right panel). B) Chemical structure of azido-VHL ligand and click reaction conditions. C) Chemical structures of 3' modified oligonucleotides (OT17- with a phosphorothioate backbone) after the click reaction with azido-VHL ligand.

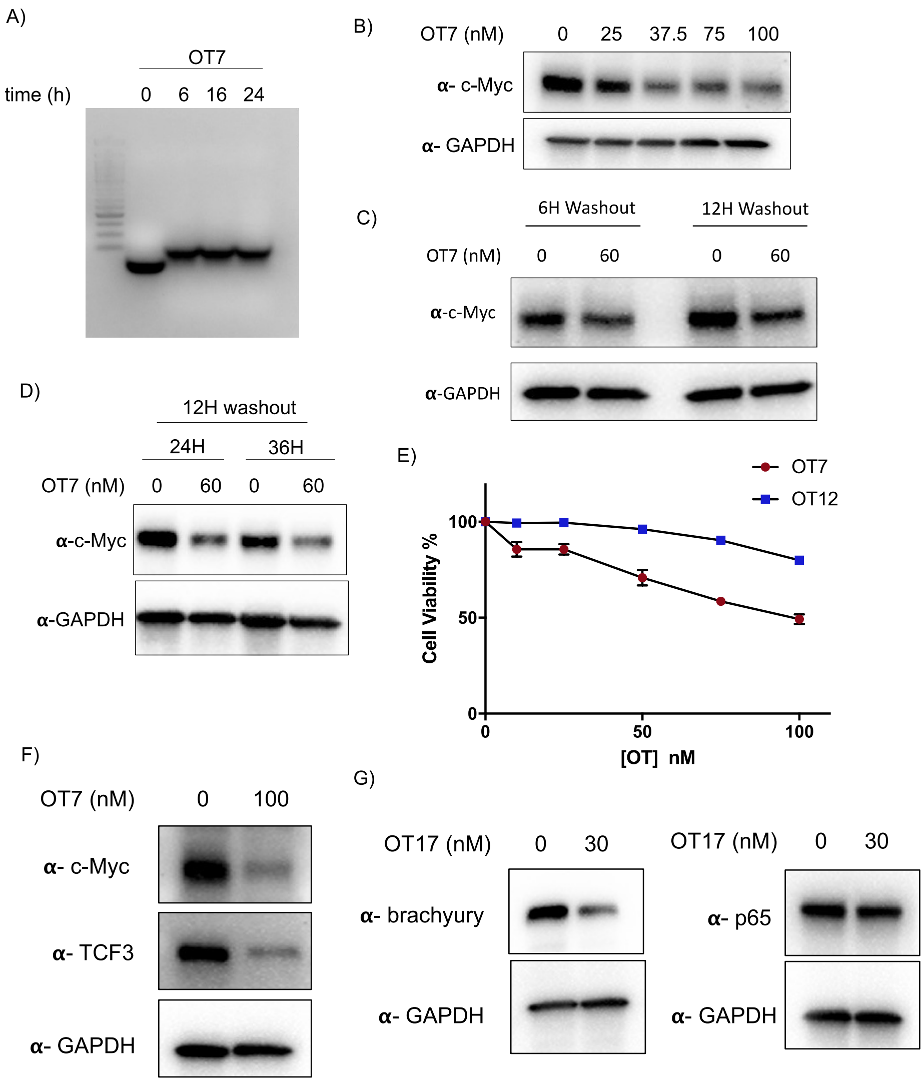

Figure S2. OligoTRAFTAC mediated c-Myc degradation. A) Electrophoretic mobility shift assay data for the click reaction. Before and after click reaction, oligonucleotides were separated from a 1.2 % agarose gel for 1 h at constant 120 mV. VHL ligand reacted oligonucleotides were shifted compared to the unreacted oligo. (n=2) B) Varying concentrations of OT7 were transfected into HEK293T cells and lysed after 20 h. Cell lysates were separated and transferred to a PVDF membrane followed by immunoblotting with antibodies against c-Myc and GAPDH. (n=2) C) After 60 nM of OT7 transfection to HEK293T cells, cell culture media was replaced with fresh medium and continued the incubation for total of 20 h prior to lysis. Lysates were probed for c-Myc and GAPDH.

(n=2) D) Similarly, HEK293T cells were subjected to a washout experiment 12 h post-transfection and lysed after 24 h and 36 h. (n=2) E) HeLa cells were seeded into 96-well plates transfected increasing concentrations of OT7 and OT12. After 48 h, cell viability was monitored using CellTiter-Glo reagent. F) Degradation of E-box binding transcription factor, TCF3. OT7 transfected cells were analyzed for c-Myc and TCF3. G) Analysis of brachyury and p65 degradation by OT17. (n=2)

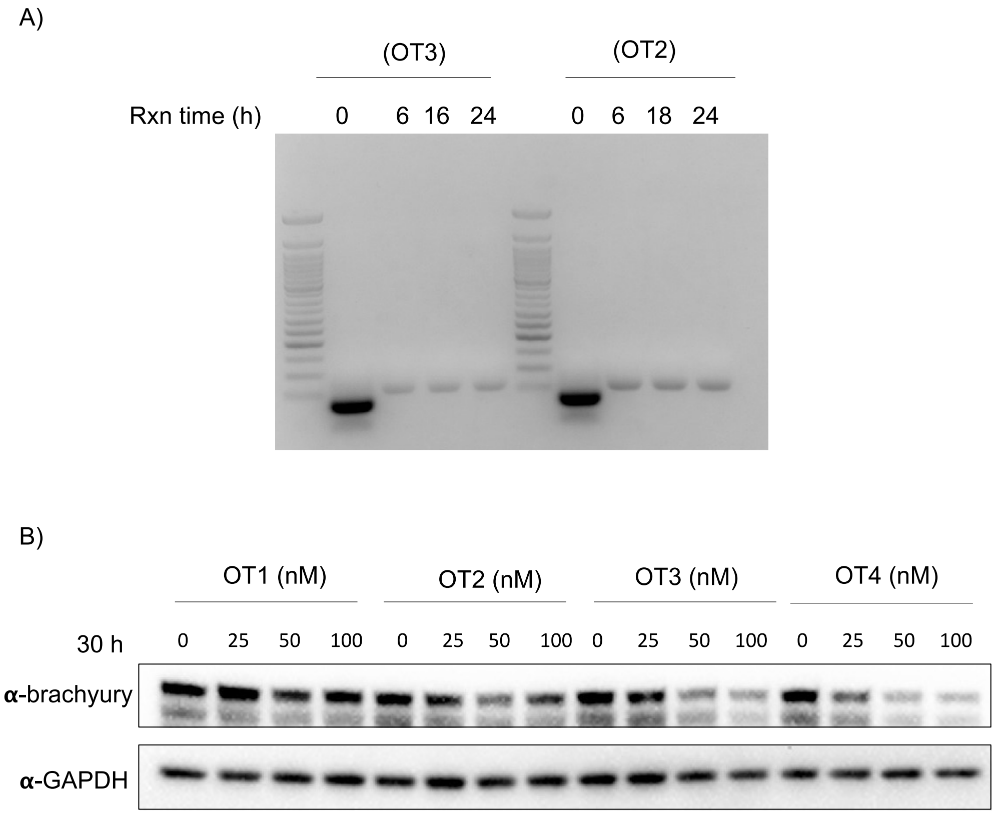

Figure S3. EMSA and brachyury-GFP degradation data. A) Click reaction mixtures with or without VHL ligand were loaded on to a 1.2% agarose gel and separated over 1 h at constant 120 mV. (n=2) B) OT1 through 4 were transfected into HEK293T cells overexpressing brachyury-GFP and lysed after 30 h. Cell lysates were subjected to SDS-PAGE and western blotting followed by probing with antibodies against brachyury and GAPDH. (n=2)

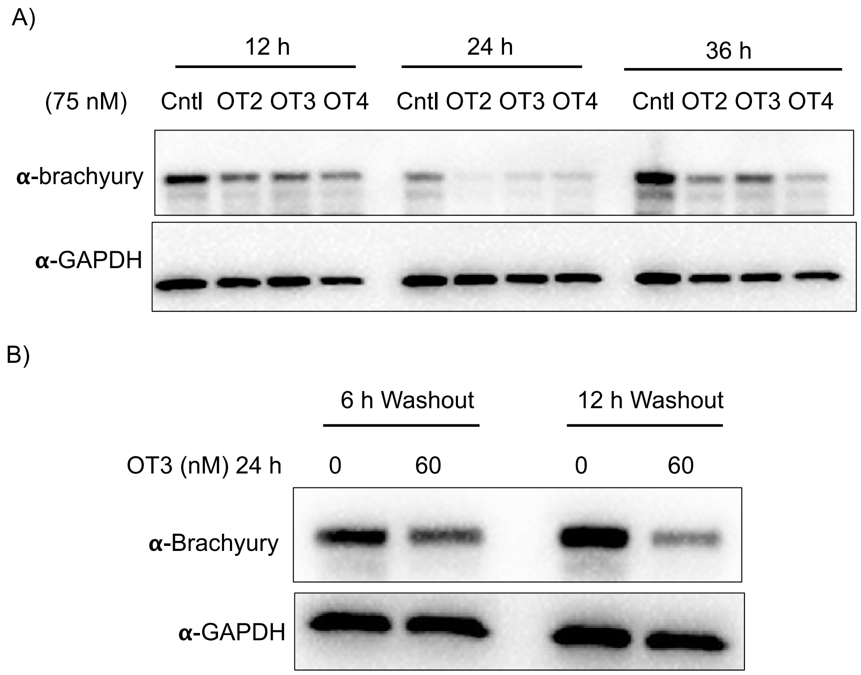

Figure S4. Time course and washout experiments for brachyury targeting OTs. A) OT2 through 4 were transfected at 75 nM into HEK293T cells overexpressing brachyury-GFP and lysed after 12h, 24 h, and 36 h. Lysates were probed for brachyury and GAPDH. (n=2) B) OT3 was transfected into HEK293T cells and washed out after 6 h and 12 h. Cell lysates were probed with antibodies against brachyury and GAPDH. (n=2)

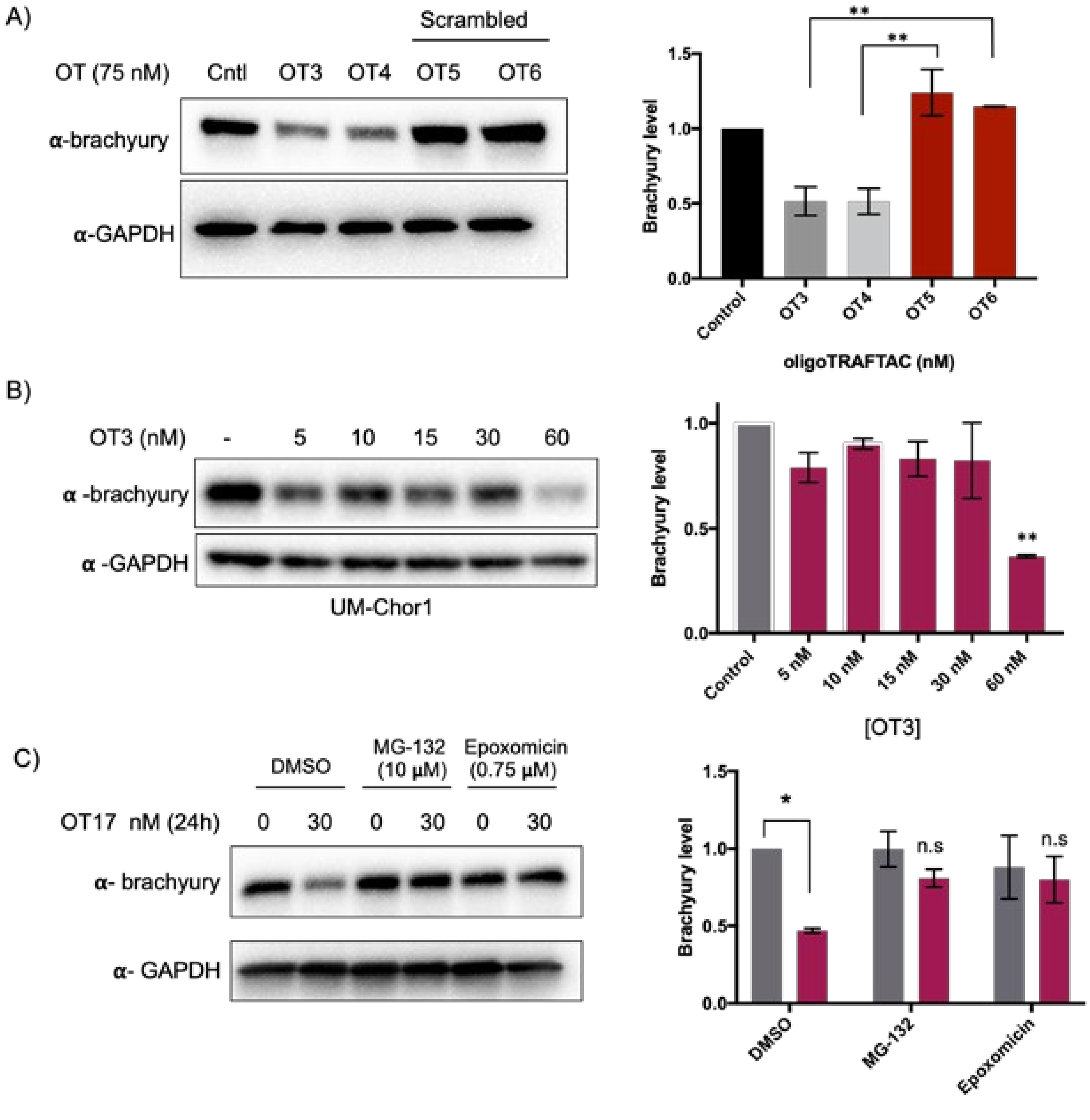

Figure S5. Brachyury degradation by oligoTRAFTACs in HEK293T and UM-Chor1 cells. A) Brachyury-GFP degradation by OTs is sequence dependent. OT3, OT4, and their scrambled OTs (OT5 and OT6) were transfected and lysed after 20 h. Cell lysates were subjected to SDS-PAGE and western blotting. Blots were probed with brachyury

and GAPDH antibodies. Quantitation of western blot bands is shown on the right. (n=2, **p<0.005) B) Increasing concentration of OT3 were transfected into UM-Chor1 cells and degradation was evaluated after 24 h. (n=2, **p<0.005) C) Proteasome dependent brachyury degradation in UM-Chor1 cells. The proteasome inhibitors, MG-132 and Epoxomicin were pre-incubated with cells prior to OT17 transfection and assessed brachyury levels after 24 h. (n=2, *p<0.05)

3. General chemistry methods

3.1 Synthesis of LC-HO-2100

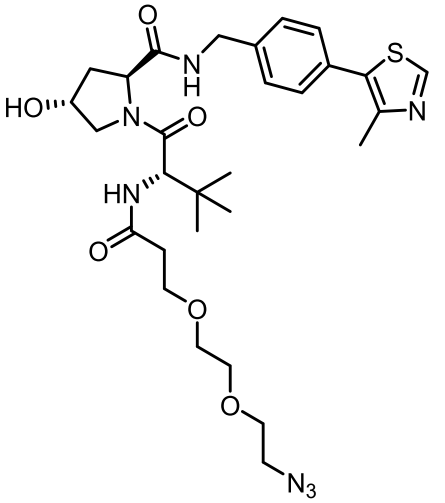

To a solution of (2S,4R)-1-[(2S)-2-amino-3,3-dimethylbutanoyl]-4-hydroxy-N-[[4-(4-methylthiazol-5-yl)phenyl]methyl]pyrrolidine-2-carboxamide hydrochloride (1.0 eq., 20 mg) in DMF (1 mL) was added (2,5-dioxopyrrolidin-1-yl) 3-[2-(2-azidoethoxy)ethoxy]propanoate (1.2 eq.), and TEA (5.0 eq.). The mixture was stirred for 1 hour at room temperature. Upon completion, the mixture was diluted with H_2O (5 mL), extracted with EtOAc (3X5 mL), dried over Na_2SO_4, concentrated under vacuum and

purified by prep-TLC. The title compound (23 mg, 71% yield) was obtained as a colorless oil.

3.2 Synthesis of LC-HO-2113

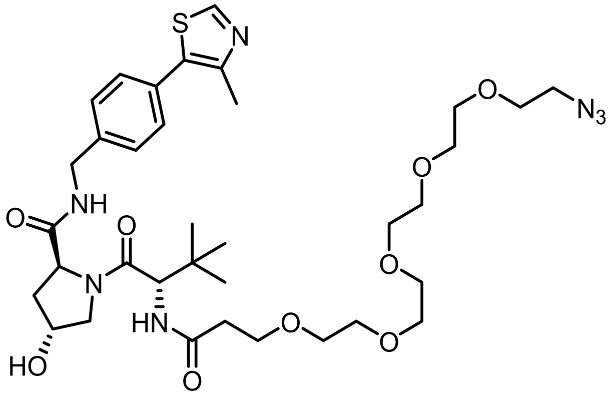

To a solution of (2S,4R)-1-[(2S)-2-amino-3,3-dimethylbutanoyl]-4-hydroxy-N-[[4-(4-methylthiazol-5-yl)phenyl]methyl]pyrrolidine-2-carboxamide hydrochloride (1.0 eq., 20 mg) in DMF (1 mL) was added(2,5-dioxopyrrolidin-1-yl)3-[2-[2-[2-[2-(2-azidoethoxy)ethoxy]ethoxy]ethoxy]ethoxy]propanoate (1.2 eq.), and TEA (5.0 eq.). The mixture was stirred for 1 hour at room temperature. Upon completion, the mixture was diluted with H_2O (5 mL), extracted with EtOAc (3X5 mL), dried over Na_2SO_4, concentrated under vacuum and purified by prep-TLC. The title compound (19 mg, 57% yield) was obtained as a colorless oil.

3.3 1H NMR and 13C NMR for LC-HO-2100

Chemical shifts are reported in δ ppm referenced to an internal $CDCl_3$ (δ 7.26 ppm) for **^{1}H NMR**, $CDCl_3$ (δ 77.00) for **^{13}C NMR.**

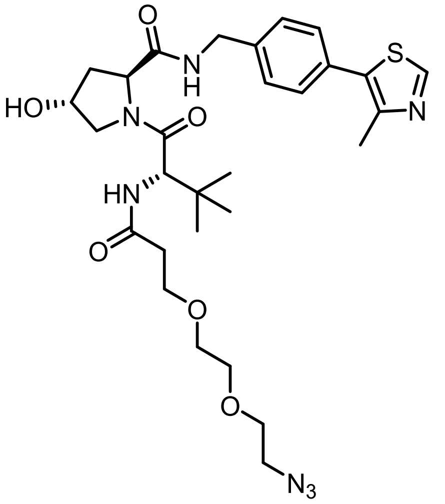

LC-HO-2100

^{1}H NMR (600 MHz, $CDCl_3$) δ 8.67 (s, 1H), 7.42 (t, *J* = 6.0 Hz, 1H), 7.36 – 7.32 (m, 4H), 6.99 (d, *J* = 8.4 Hz, 1H), 4.70 – 4.67 (m, 1H), 4.54 – 4.51 (m, 1H), 4.47 – 4.46 (m, 1H), 4.11 (d, *J* = 11.4 Hz, 1H), 3.73 – 3.69 (m, 3H), 3.64 (s, 3H), 3.65 – 3.62 (m, 3H), 3.59 (dd, *J* = 10.8, 3.6 Hz, 1H), 3.37 – 3.35 (m, 2H), 2.50 (s, 3H), 2.53 – 2.47 (m, 3H), 2.13 – 2.09 (m, 3H), 0.93 (s, 9H).

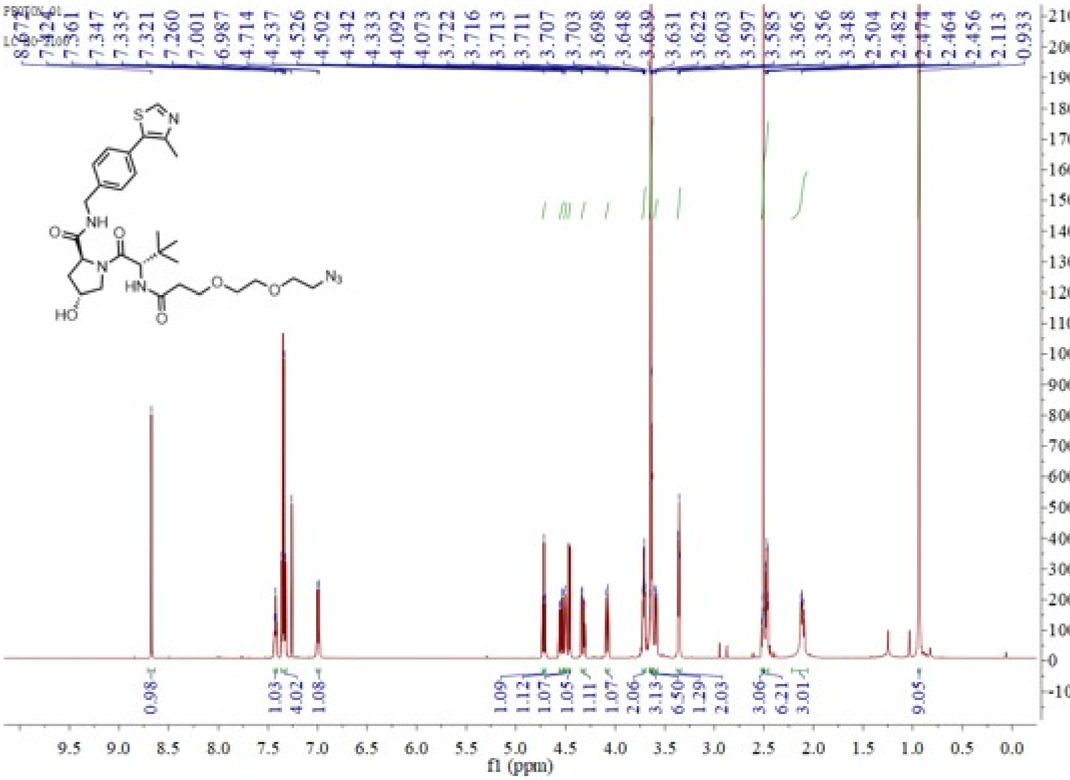

^{13}C NMR (150 MHz, $CDCl_3$) δ 172.07, 171.71, 170.76, 150.28, 148.40, 138.10, 131.55, 130.87, 129.45, 128.06, 70.45, 70.42, 70.02, 69.97, 67.13, 58.37, 57.67, 56.58, 50.52, 43.17, 36.65, 35.82, 34.78, 26.35, 16.02.

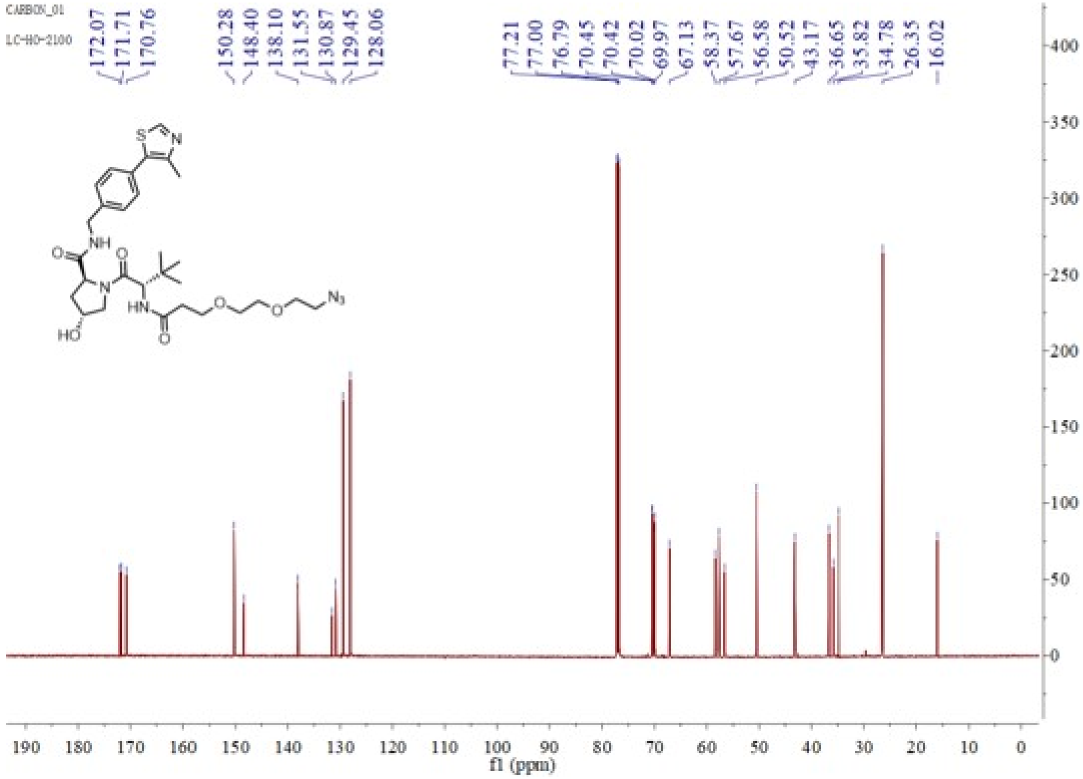

3.4 1H NMR and 13C NMR for LC-HO-2113

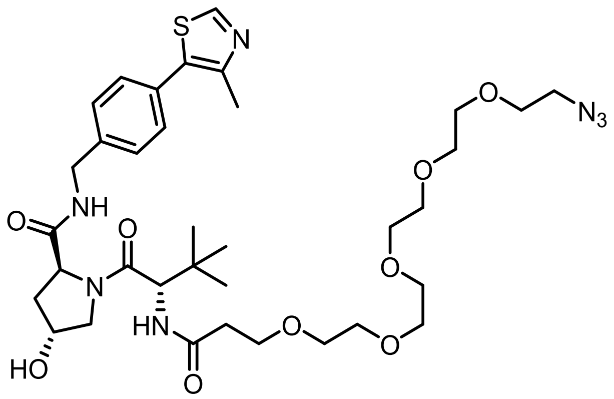

LC-HO-2113

^{1}H NMR (600 MHz, $CDCl_3$) δ 8.66 (s, 1H), 7.47 (s, 1H), 7.34 – 7.33 (m, 4H), 7.04 –7.03 (m, 1H), 4.71 – 4.67 (m, 1H), 4.55 – 4.50 (m, 1H), 4.49 – 4.44 (m, 2H), 4.33 – 4.29 (m, 1H), 4.05 (d, *J* = 10.8 Hz, 1H), 3.69– 3.66 (m, 3H), 3.65 – 3.58 (m, 17H), 3.36 – 3.35 (m, 2H), 2.49 – 2.48 (m, 4H), 2.48 – 2.38 (m, 4H), 2.13 – 2.07 (m, 1H), 0.92 (s, 9H).

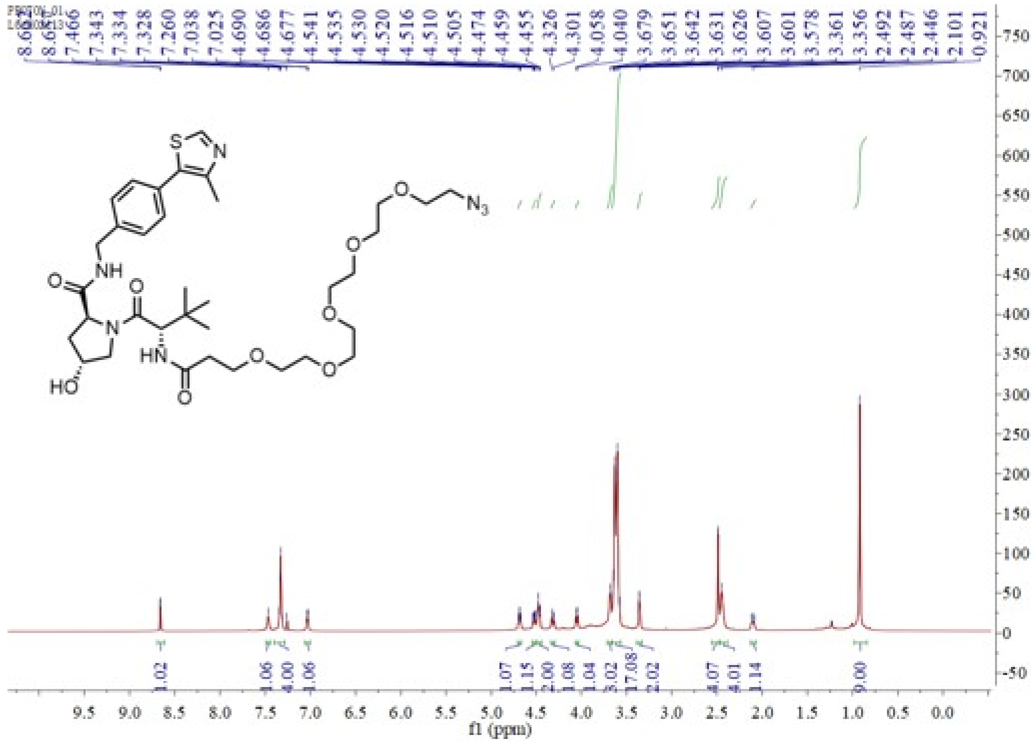

^{13}C NMR (150 MHz, $CDCl_3$) δ 172.01, 171.59, 170.91, 150.25, 148.34, 138.16, 131.55, 130.76, 129.39, 127.99, 70.59, 70.54, 70.50, 70.44, 70.41, 70.37, 70.32, 69.97, 69.95, 67.04, 58.45, 57.65, 56.62, 50.59, 43.08, 36.61, 36.03, 34.87, 26.33, 15.99.

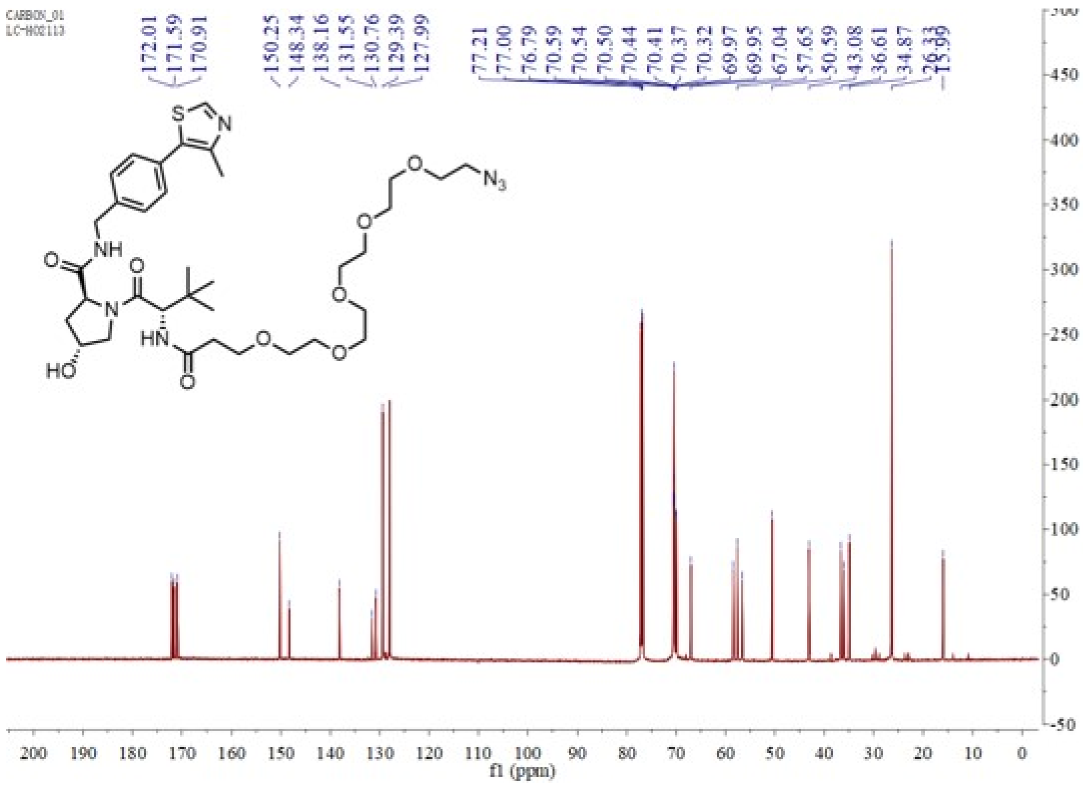

4. Mass spectra for oligonucleotides

Brachyury (Expected mass-11384.51)

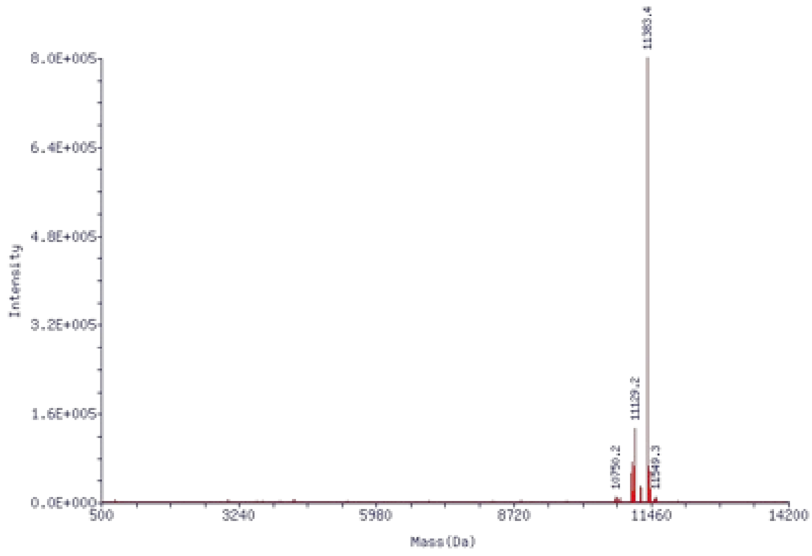

c-Myc (Expected mass-9396.17)

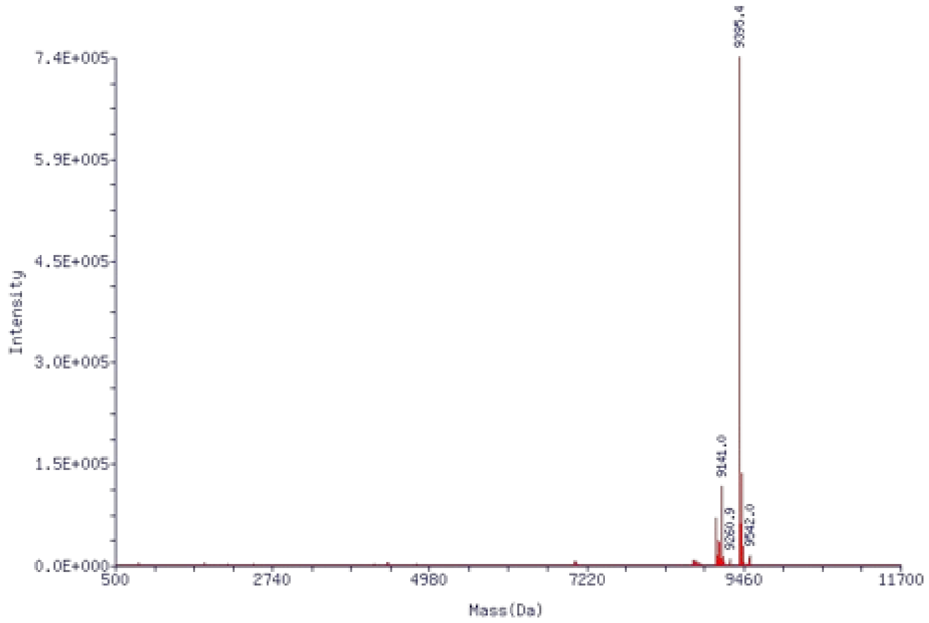

Brachyury-Phosphorothioate (Expected mass-11655.01)

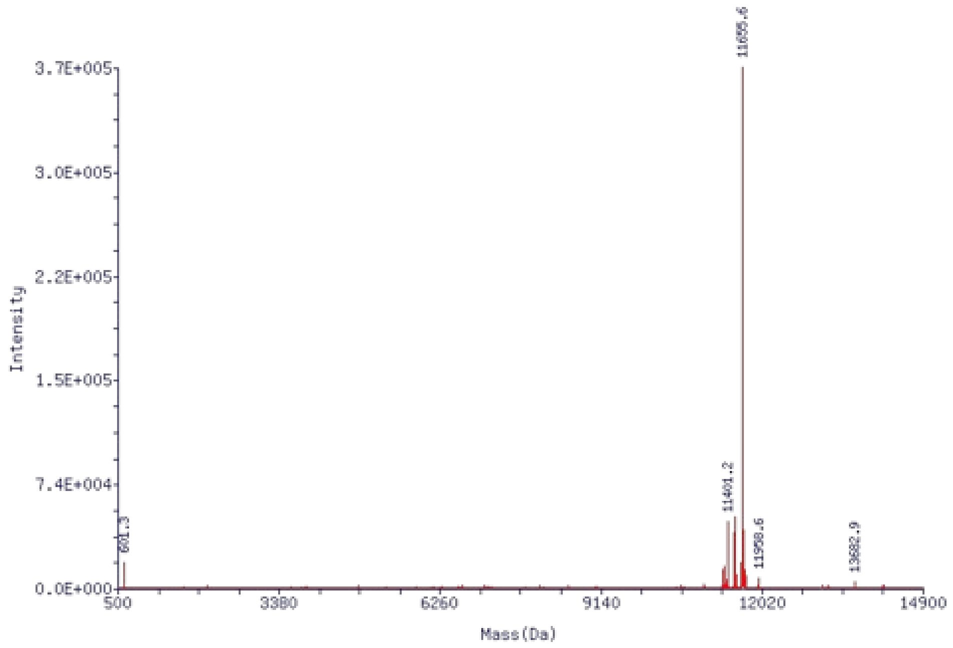

Scrambled-brachyury (Expected mass-11384.51)

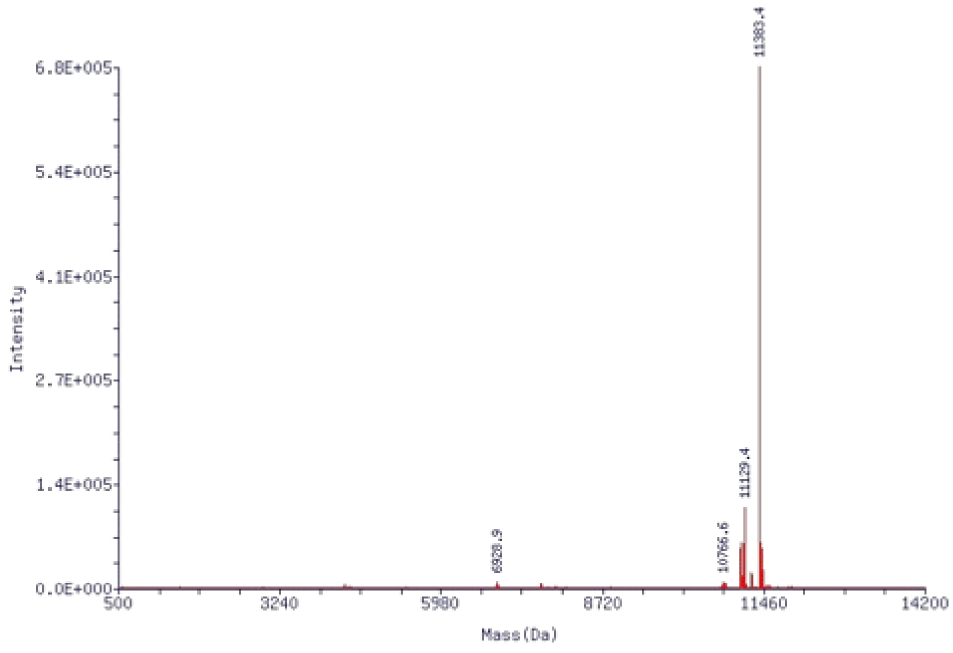

Scrambled-Myc (Expected mass-9396.17)

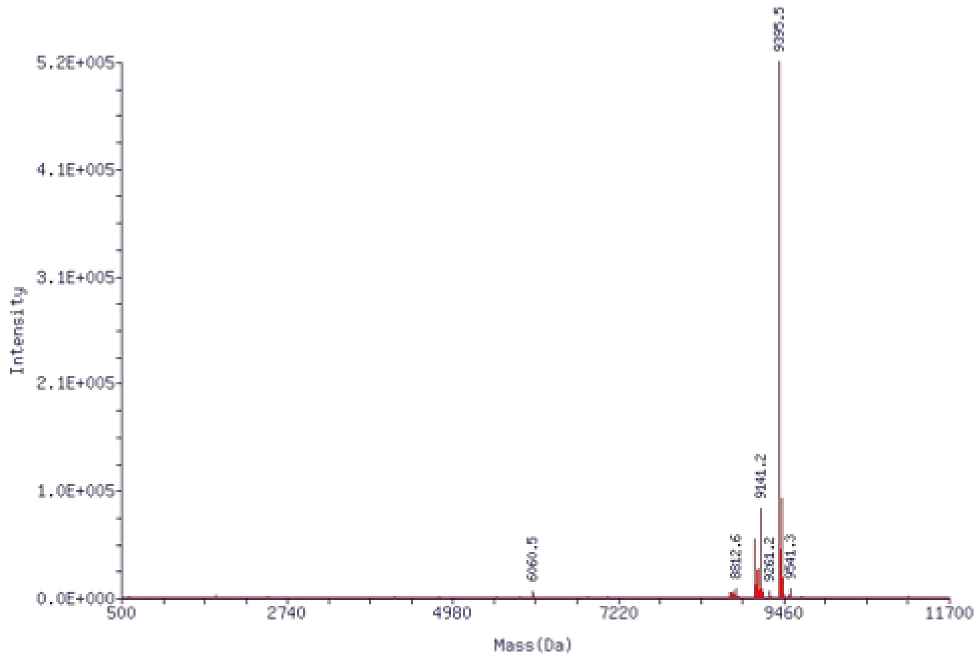

5. Oligonucleotide sequences

Table S1. Oligonucleotide sequence, modification site and linker lengths of oligoTRAFTACs used in this study

OT#	Target	Oligonucleotide sequence 5' - 3'	Modification	Modification site	Linker PEG, n=
OT1	Brachyury	CTTTCC**AATTTCACACCTAGGTGTGAAATT** GGGGAC	Alkyne	5'	5
OT2	Brachyury	CTTTCC**AATTTCACACCTAGGTGTGAAATT** GGGGAC	Alkyne	5'	2
OT3	Brachyury	CTTTCC**AATTTCACACCTAGGTGTGAAATT** GGGGAC	Alkyne	3'	5
OT4	Brachyury	CTTTCC**AATTTCACACCTAGGTGTGAAATT** GGGGAC	Alkyne	3'	2
O5	Scrambled	ACGAGACGAGCTCTTAAGTTCCTGGCGTAC TTATAT	Alkyne	5'	2
OT6	Scrambled	ACGAGACGAGCTCTTAAGTTCCTGGCGTAC TTATAT	Alkyne	3'	5
OT7	c-Myc	TGGGAG **CACGTGGTTGCCACGTG** GTTGGG	Alkyne	5'	2
OT10	c-Myc	TGGGAG **CACGTGGTTGCCACGTG** GTTGGG	Alkyne	3'	2
OT12	Scrambled	GTATGT GAGCGGTGGTGGCGTGC CAGCGT	Alkyne	5'	2

OT17	Brachyury	C*T*T*T*C*C***A*A*T*T*T*C*A*C*A*C*C*T*A*** **G*G*T*G*T*G*A*A*A*T*T*** G*G*G*G*A*C	Alkyne	3’	5
OT20	Scrambled	A*C*G*A*G*A*C*G*A*G*C*T*C*T*T*A*A*G*T*T* C*C*T*G*G*C*G*T*A*C* T*T*A*T*A*T	Alkyne	3’	5

Table S2. Oligonucleotide sequence, modification and modification site of the oligonucleotides used for biotin pull-down experiments

Target	Oligonucleotide sequence 5' - 3'	Modification	Modification site
c-Myc	TGGGAG **CACGTGGTTGCCACGTG** GTTGGG	Biotin	5'
Scrambled	GTATGT GAGCGGTGGTGGCGTGC CAGCGT	Biotin	5'
Brachyury	CTTTCC**AATTTCACACCTAGGTGTGAAATT** GGGGAC	Biotin	3'
Scrambled	ACGAGACGAGCTCTTAAGTTCCTGGCGTAC TTATAT	Biotin	3'

6. Original blots

Figure 2

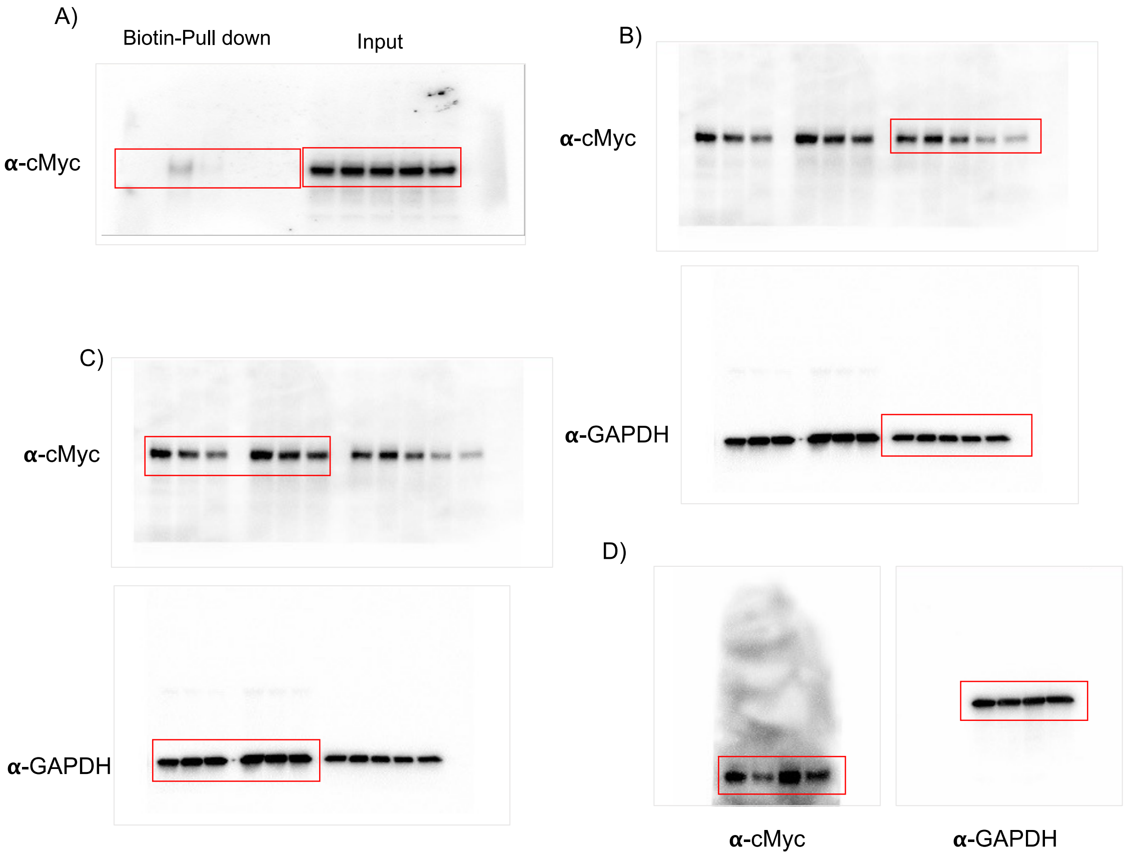

Figure 3

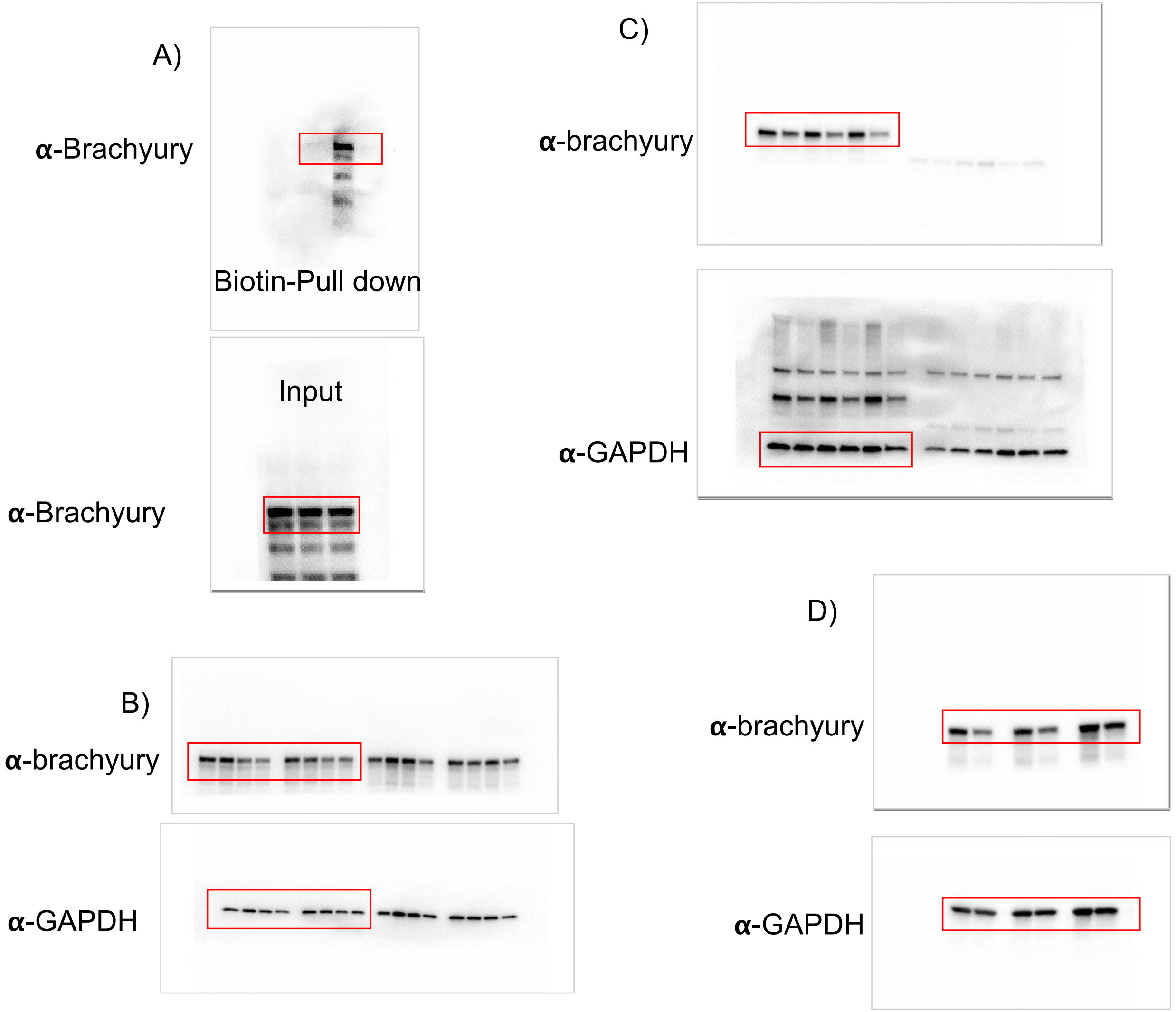
A)
α-Brachyury
Biotin-Pull down
Input
α-Brachyury
B)
α-brachyury
α-GAPDH
C)
α-brachyury
α-GAPDH
D)
α-brachyury
α-GAPDH

Figure 4

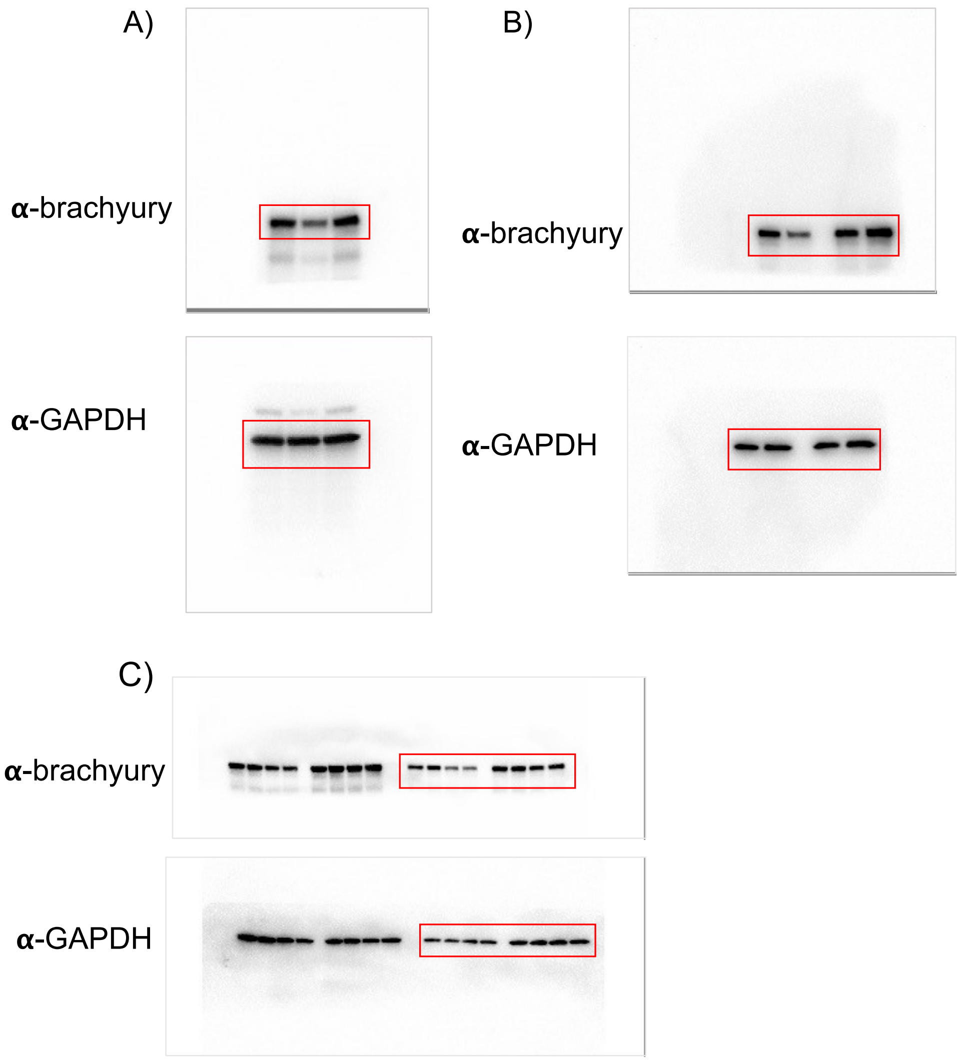
A)
α-brachyury
α-GAPDH
B)
α-brachyury
α-GAPDH
C)
α-brachyury
α-GAPDH

Figure 5

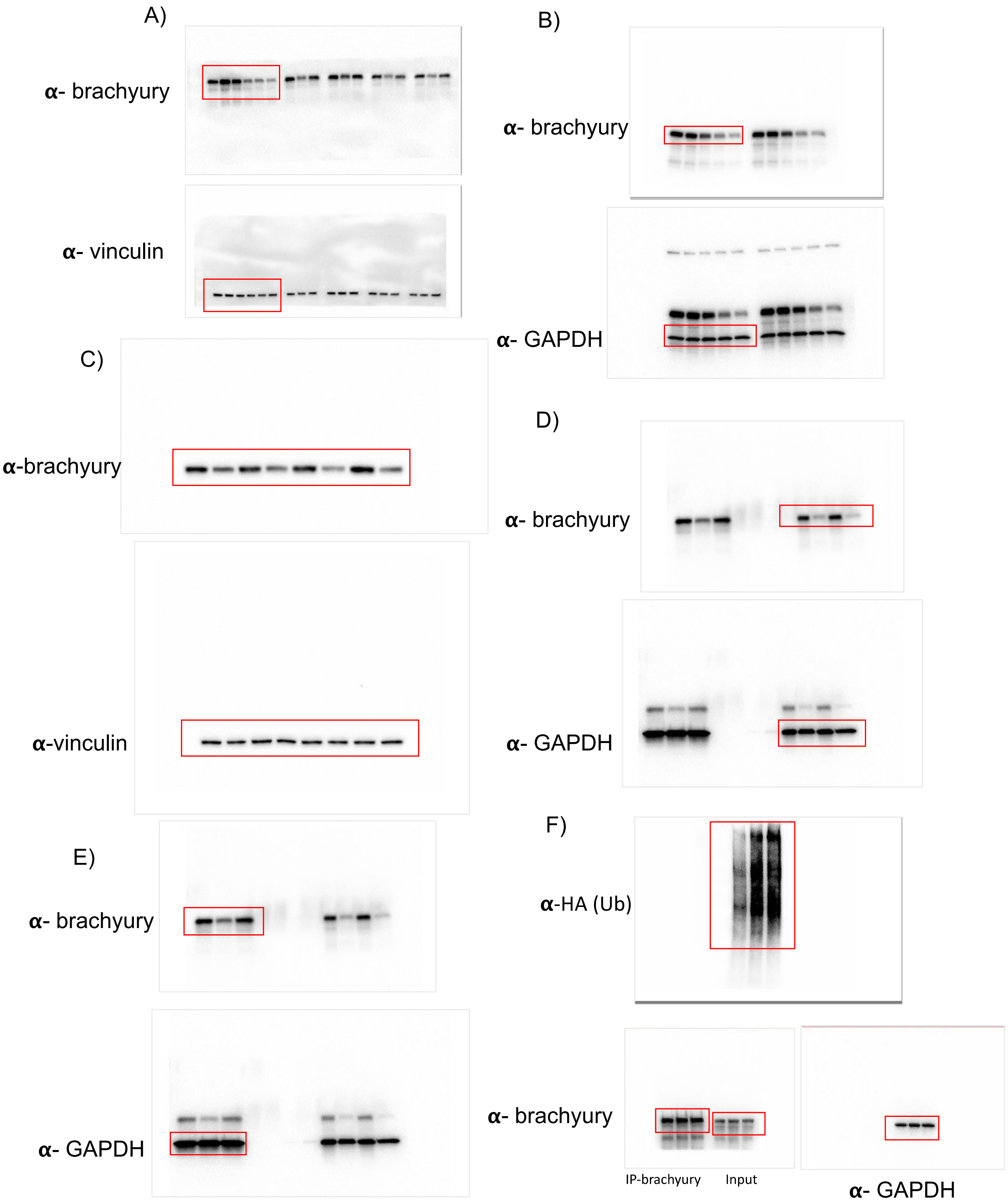
A)
α- brachyury
α- vinculin
B)
α- brachyury
α- GAPDH
C)
α-brachyury
α-vinculin
D)
α- brachyury
α- GAPDH
E)
α- brachyury
α- GAPDH
F)
α-HA (Ub)
α- brachyury
IP-brachyury
Input
α- GAPDH

Figure 6

D)

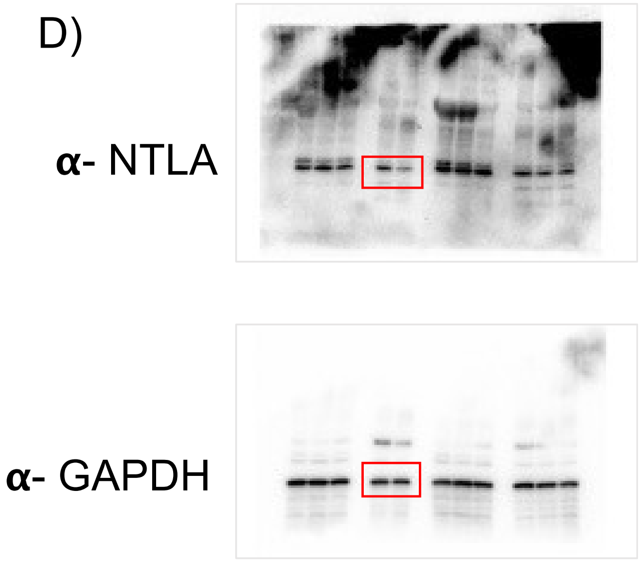

Supporting Figure 2

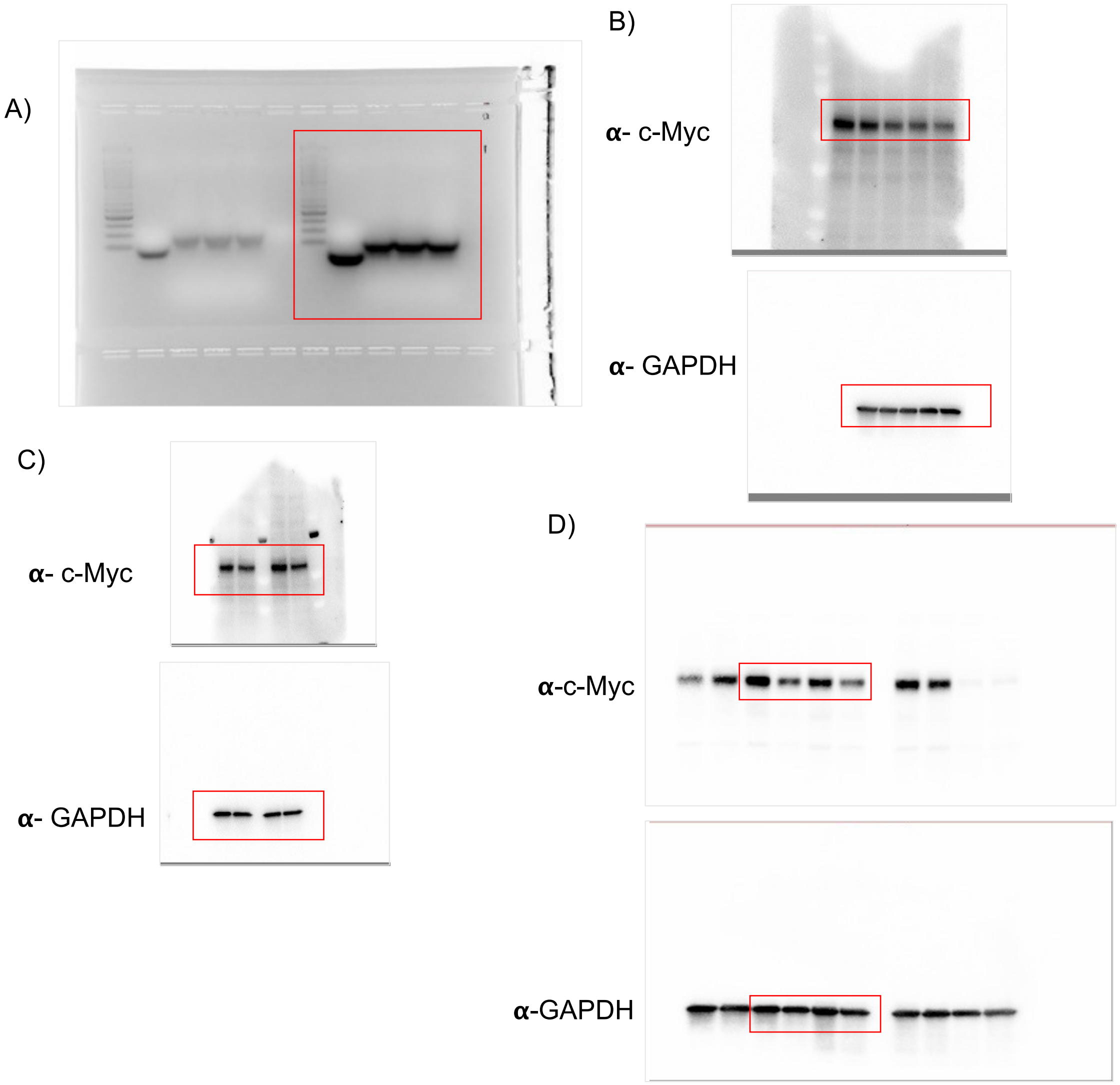

Supporting Figure 2, Continue

F)

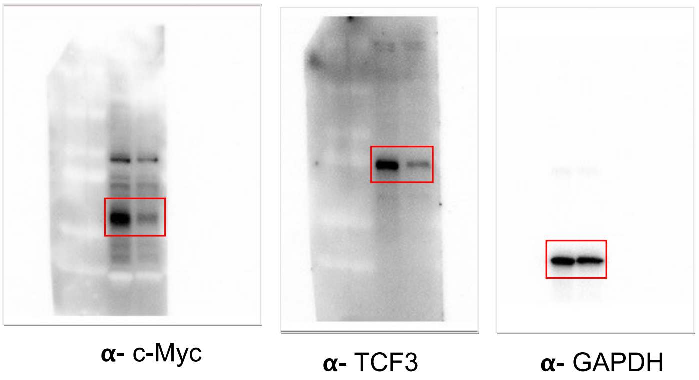

G)

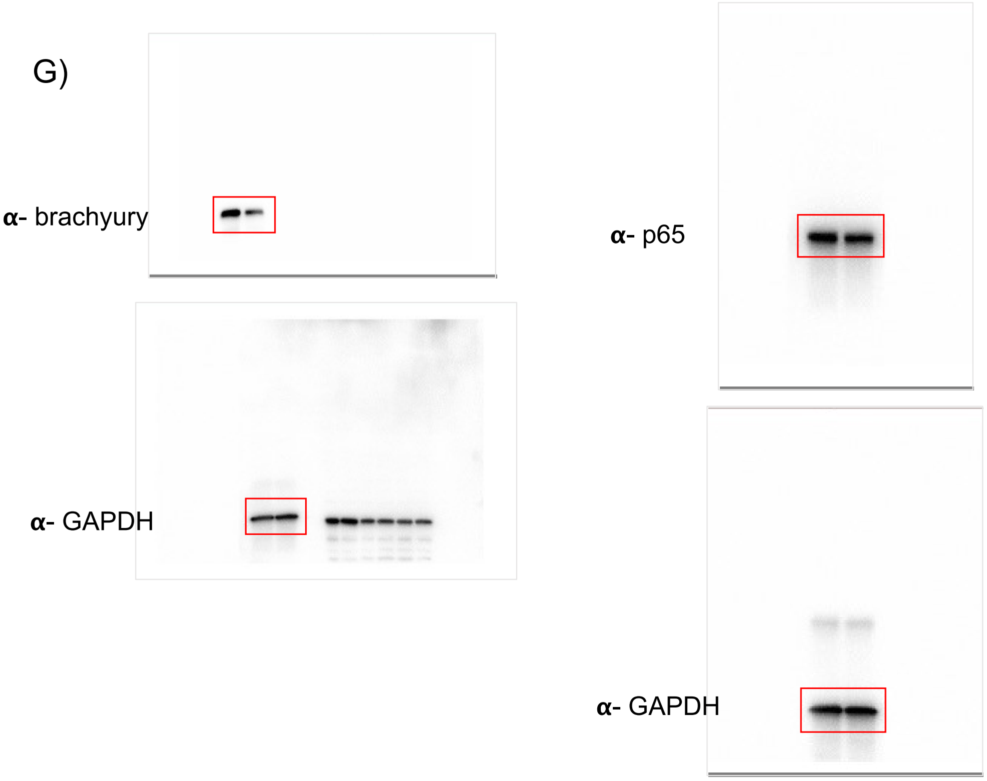

Supporting Figure 3

A)

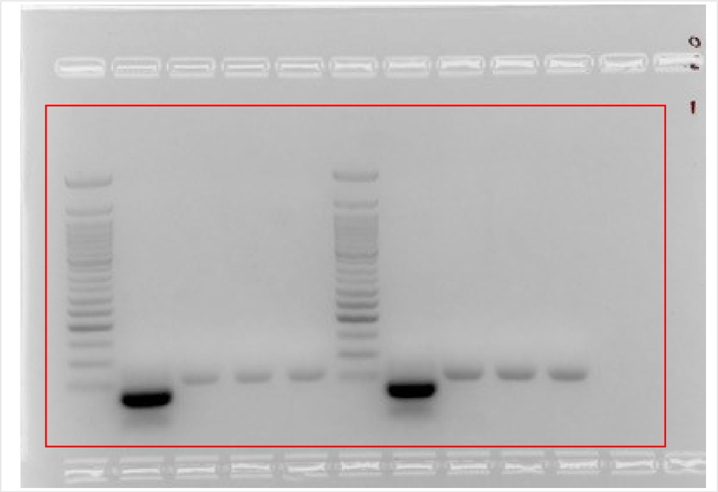

B)

α-brachyury

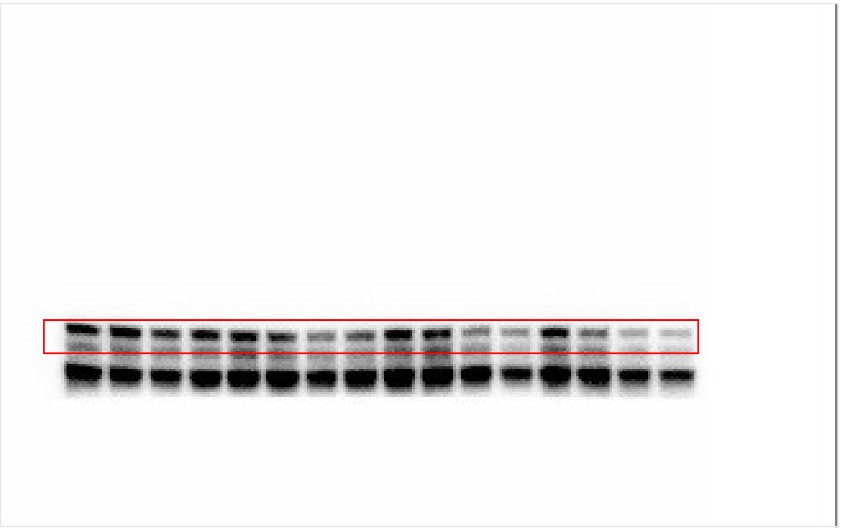

α-GAPDH

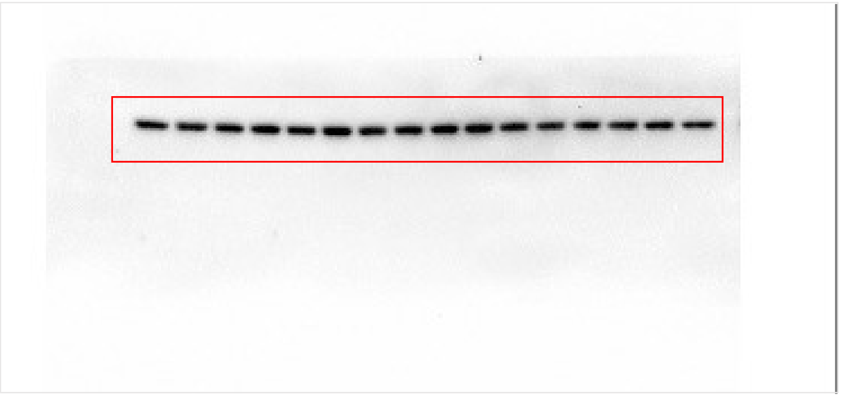

Supporting Figure 4

Supporting Figure 5

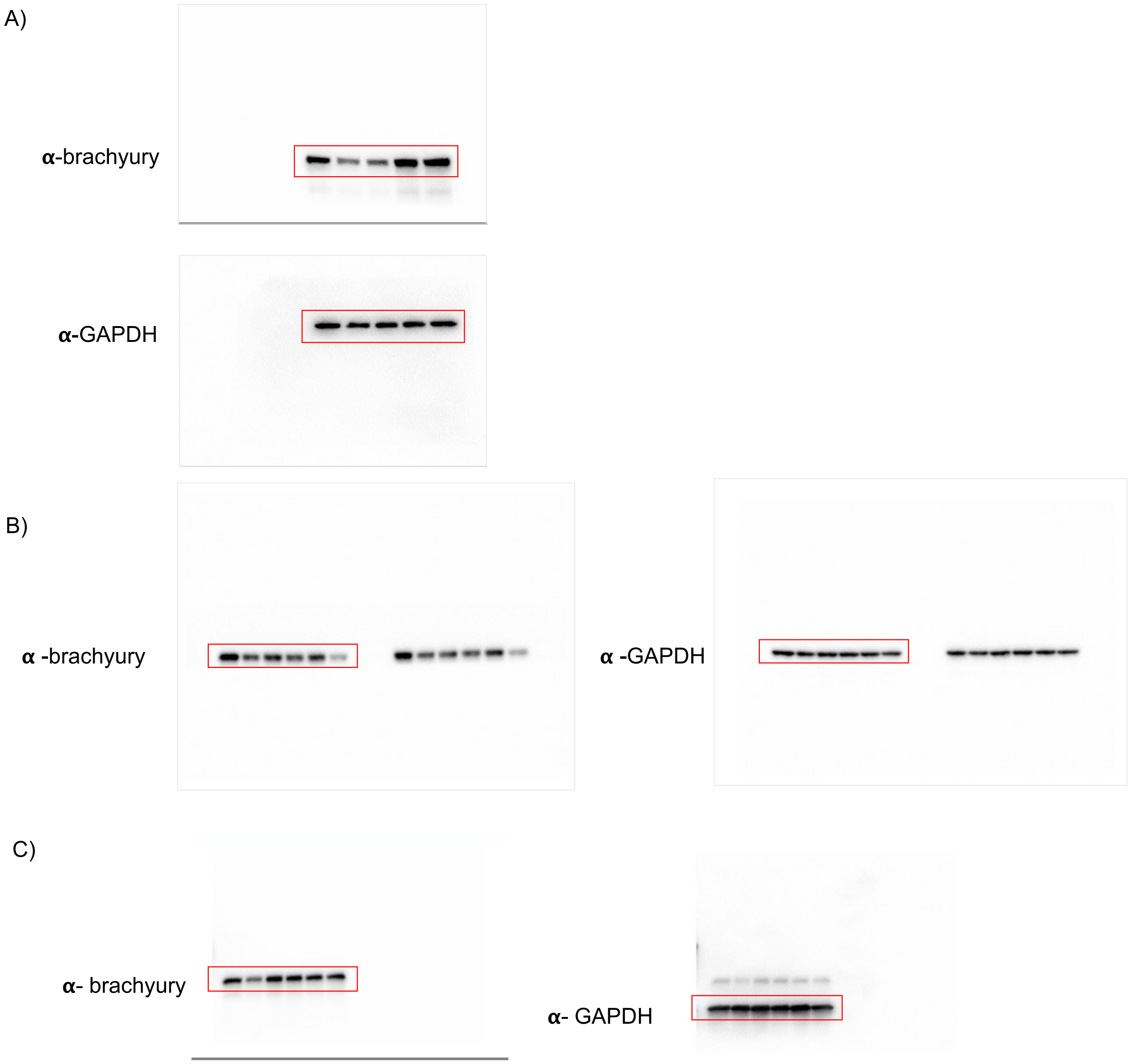

7. References

1. C. Wilson, S. High, B. McCluskey, A. Amores, Y. Yan, T. Titus, J. Anderson, P. Batzel, M. Carvan, M. Schartl and J. Postlethwait, *Genetics*, 2014, 198.

2. D. Kimelman, *Developmental Dynamics*, 2016, 245, 874-880.
